# Targeting sphingolipid metabolism in chronic lymphocytic leukemia

**DOI:** 10.1007/s10238-024-01440-x

**Published:** 2024-07-30

**Authors:** Flora Nguyen Van Long, Trang Le, Patrick Caron, Délya Valcourt-Gendron, Roxanne Sergerie, Isabelle Laverdière, Katrina Vanura, Chantal Guillemette

**Affiliations:** 1 Centre Hospitalier Universitaire (CHU) de Québec Research Center, Faculty of Pharmacy and Université Laval Cancer Research Center, Université Laval, R4701.5, 2705 Blvd Laurier, Quebec, QC G1V 4G2 Canada; 2 Division of Haematology and Haemostaseology, Department of Medicine I, Medical University of Vienna, Währinger Gürtel 18-20, 1090 Vienna, Austria; 3 Canada Research Chair in Pharmacogenomics, Quebec, Canada

**Keywords:** Chronic lymphocytic leukemia, Sphingolipids, Lipidomics, Ibrutinib, Drug target

## Abstract

Elevated levels of circulating C16:0 glucosylceramides (GluCer) and increased mRNA expression of UDP-glucose ceramide glycosyltransferase (*UGCG*), the enzyme responsible for converting ceramides (Cer) to GluCer, represent unfavorable prognostic markers in chronic lymphocytic leukemia (CLL) patients. To evaluate the therapeutic potential of inhibiting GluCer synthesis, we genetically repressed the UGCG pathway using in vitro models of leukemic B cells, in addition to UGCG pharmacological inhibition with approved drugs such as eliglustat and ibiglustat, both individually and in combination with ibrutinib, assessed in cell models and primary CLL patient cells. Cell viability, apoptosis, and proliferation were evaluated in vitro, and survival and apoptosis were examined ex vivo. UGCG inhibition efficacy was confirmed by quantifying intracellular sphingolipid levels through targeted lipidomics using mass spectrometry. Other inhibitors of sphingolipid biosynthesis pathways were similarly assessed. Blocking UGCG significantly decreased cell viability and proliferation, highlighting the oncogenic role of UGCG in CLL. The efficient inhibition of UGCG was confirmed by a significant reduction in GluCer intracellular levels. The combination of UGCG inhibitors with ibrutinib demonstrated synergistic effect. Inhibitors that target alternative pathways within sphingolipid metabolism, like sphingosine kinases inhibitor SKI-II, also demonstrated promising therapeutic effects both alone and when used in combination with ibrutinib, reinforcing the oncogenic impact of sphingolipids in CLL cells. Targeting sphingolipid metabolism, especially the UGCG pathway, represents a promising therapeutic strategy and as a combination therapy for potential treatment of CLL patients, warranting further investigation.

**Supplementary Information:**

The online version contains supplementary material available at 10.1007/s10238-024-01440-x.

## Introduction

Bioactive lipid molecules, particularly sphingolipids, have gained increasing attention in cancer biology for their involvement in cellular signaling. These lipid molecules play crucial roles in regulating cell proliferation, apoptosis, migration, and inflammation, all of which are processes dysregulated in cancer [1, 2]. Targeting sphingolipid metabolism and signaling pathways has emerged as a promising strategy for cancer therapy. Small-molecule inhibitors of enzymes involved in sphingolipid metabolism, as well as agonists and antagonists of sphingosine-1-phosphate (S1P) receptors, are being actively investigated as potential anticancer agents [3, 4].

Few reports have emphasized alterations in sphingolipid metabolism in chronic lymphocytic leukemia (CLL) [5–12]. Significant changes in the levels of ceramides (Cer) and glucosylceramides (GluCer) were observed between CLL patients compared to healthy individuals [8, 12]. Moreover, differences in Cer and GluCer levels have been observed in CLL patients with aggressive disease characteristics, such as the presence of unmutated immunoglobulin heavy-chain variable (IGHV) genes compared to those with a favorable prognosis presenting mutated IGHV, indicating their potential significance in both CLL development and progression [5, 6, 8, 12]. In multivariate analyses, C16:0 GluCer emerged as an independent prognostic marker, associated with shorter treatment-free survival [5]. In addition, increased leukemic mRNA expression of UDP-glucose ceramide glycosyltransferase (*UGCG*), the enzyme responsible for converting Cer to GluCer (Supplementary Fig. 1), has been found to be significantly associated with poor prognosis [5]. These findings emphasize the critical role of UGCG and GluCer metabolism in CLL pathogenesis, which was corroborated by the pro-proliferative effect of C16:0 GluCer in leukemic cell models [5, 13]. The data thus imply the therapeutic potential of targeting UGCG and GluCer levels in CLL. Another relevant observation relates to sphinganine, shown to exert pro-apoptotic effect in leukemic cell models [5]. Elevated circulating sphinganine levels were significantly associated with improved survival of CLL patients, suggesting that enzymatic pathways involved in sphinganine metabolism might also serve as potential target for therapeutic intervention (Supplementary Fig. 1).

**Fig. 1:**
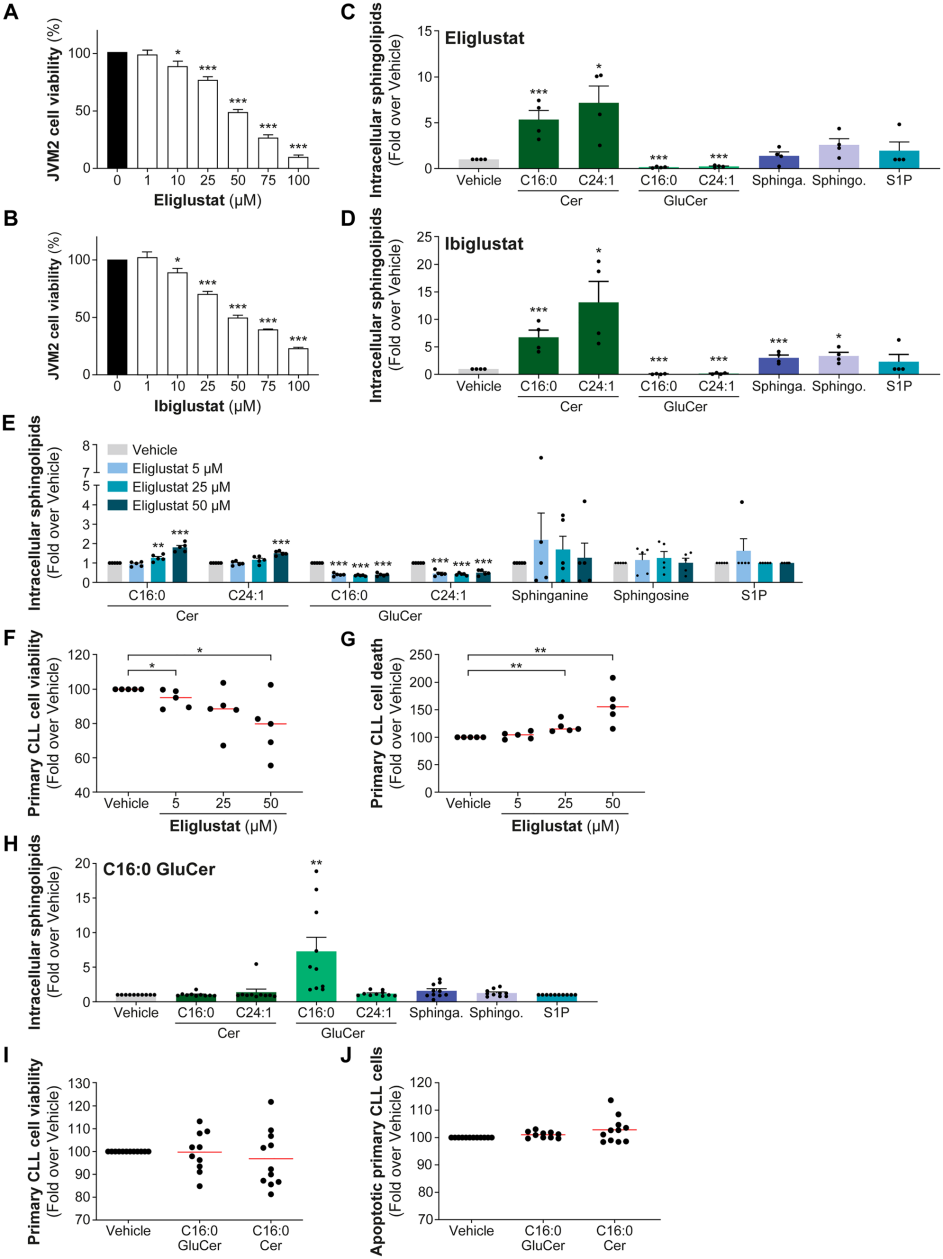
Inhibiting UGCG pharmacologically decreased cell viability in vitro and in primary CLL. UGCG inhibitors (UGCGi), eliglustat and ibiglustat, target the conversion between ceramides (Cer) to glucosylceramides (GluCer, Supplementary Fig. 1). JVM2 cells were treated with increasing concentrations of eliglustat (**A**) or ibiglustat (**B**) for 72 h, and cell viability was measured by MTS assay. Data are compared to untreated cells (vehicle). The efficacy and specificity of UGCG inhibition in JVM2 cells using IC_50_ concentrations of UGCGi for 72 h corresponding to 42 µM eliglustat (**C**) and 48 µM ibiglustat (**D**) were assessed by quantifying intracellular sphingolipid concentrations using a targeted lipidomics approach. Absolute quantification of sphingolipid levels is represented in Supplementary Fig. 2. Data are expressed as the mean ± standard error from four independent experiments. (**E**–**G**) Peripheral blood mononuclear cells from five CLL patients were treated with 5, 25, and 50 µM eliglustat for 46 h, treatments efficiency was assessed by mass spectrometry (**E**). Effects of eliglustat treatments on primary CLL cell viability by CellTiter-Blue assay (**F**) and cell death by FACS using DAPI-positive cells (**G**) are shown. (**H**–**J**) Twelve primary cells from CLL patients were treated with 10 µM C16:0 GluCer (1:1 CHCL_3_/MeOH) or C16:0 Cer (1:1 CHCL_3_/MeOH) for 22 h. Ten out of 12 patients presented enhanced intracellular concentration of C16:0 GluCer confirmed by MS (**H**). In these conditions, cell viability (**I**) measured by CellTiter-Blue assay and apoptosis (**J**) assessed by FACS using DAPI and Annexin V staining is reported. CLL cells that were Annexin V positive were considered as apoptotic. Data are expressed as the mean ± standard error. Red line corresponds to the mean values. *P < .05; **P < .01; and ***P < .001. Sphinga.: sphinganine; sphingo.: sphingosine; and S1P: sphingosine-1-phosphate

To date, only a limited number of studies have investigated the effects of sphingolipid inhibitors in the context of CLL. Gerrard et al. demonstrated that inhibiting UGCG sensitized CLL cells to active agents used in CLL such as fludarabine and chlorambucil, suggesting a potential role for UGCG inhibition in enhancing the efficacy of conventional chemotherapy [14]. This is consistent with another report conducted in CLL patients, which revealed that resistance to fludarabine was linked to overexpression of UGCG and that treatment with a UGCG inhibitor (UGCGi) restored sensitivity to fludarabine-resistant cells in vitro [13]. Schwamb et al. reported that treatment with UGCGi sensitized CLL cells in which B cell receptor was engaged by immunoglobulin M stimulation, to a targeted agent, specifically a BCL-2 inhibitor. However, UGCGi as a monotherapy did not significantly affect the viability of primary CLL cells [6]. Two studies reported that inhibiting sphingosine kinases (SPHK), responsible for the conversion of S1P from sphingosine, enhanced the effect of several therapeutic agents such as fludarabine and ibrutinib [10] and sensitized resistant cells to venetoclax induced by the activation of lymphocytes T cells [15]. These findings collectively suggest that targeting sphingolipid metabolism may hold promise as a therapeutic strategy in CLL, particularly in combination with both chemotherapy and targeted therapy, to overcome drug resistance and enhance treatment efficacy.

The aim of this study was to evaluate whether inhibiting GluCer production by targeting UGCG, both pharmacologically and genetically, could impact leukemic cells survival, and in combination with ibrutinib targeting Bruton tyrosine kinase (BTK) used in the first-line setting for CLL. Additional inhibitors of sphingolipids were assessed prompted by the association of circulating sphinganine levels with treatment-free survival outcome in CLL patients [5]. Our investigations were focused on leukemic B cell models and primary cells from CLL patients and the use of a targeted lipidomics approach to validate the effects of approved sphingolipid inhibitory drugs on the intracellular sphingolipid levels. Our findings support the notion that targeting sphingolipids, namely, by inhibiting UGCG or sphingosine kinases, may hold promise as a therapeutic strategy in CLL.

## Material and methods

### Chemicals

All chemicals used in this study are described in Supplementary Material and Methods.

### CLL patient cells

Cryopreserved peripheral blood mononuclear cells (PBMCs) from 17 CLL patients diagnosed between 1987 and 2011 at Vienna General Hospital were studied. Patients’ characteristics are outlined in Supplementary Table 1. PBMCs were cultured in RPMI 1640 GlutaMAX medium supplemented with 25 mM HEPES (N-2-hydroxyethylpiperazine-N'-2-ethanesulfonic acid), 1 mM sodium pyruvate, and 10% heat-inactivated fetal bovine serum. All components for cell culture were purchased from Thermo Fischer Scientific (Waltham, MA, USA).

PBMCs (Supplementary Table 2) were plated at a density of 3.2 × 10^6^ cells/mL in 6-well plates and treated with 5 µM, 25 µM, and 50 µM of UGCGi, eliglustat or SPHK inhibitor (SPHKi), SKI-II or vehicle (< 0.1% DMSO) for 46 h. For combined treatment with ibrutinib, PBMCs were treated with either 50 µM eliglustat or SKI-II with 0.3 µM ibrutinib or with vehicle for 46 h. C16:0 GluCer and C16:0 Cer were dissolved in a vehicle of CHCL_3_/MeOH (1:1), and sphinganine, sphingosine, and sphingosine-1-phosphate (S1P) were prepared in MeOH. All sphingolipids were dissolved in a working solution of 10 mM. To assess the effects of sphingolipids, PBMCs (Supplementary Table 3) were plated at a density of 3.3 × 10^6^ cells/mL in 12-well plates and treated with 10 μM of sphingolipids or the corresponding vehicle (0.1% CHCL_3_/MeOH or 0.1% MeOH) for 22 h. Cell viability was assessed using the CellTiter-Blue Cell Viability Assay (Promega), and apoptosis was determined using DAPI (4',6-diamidino-2-phenylindole, Sigma-Aldrich) and APC Annexin V (BioLegend, San Diego, CA, USA) staining as described previously [16]. The study was performed in accordance with the Helsinki Declaration and was approved by local Ethical Research Committees of the Medical University of Vienna (Ethics vote 2176/2017) and CHU de Québec–Université Laval (2015–1205).

### Targeted lipidomic assay

Intracellular sphingolipids concentrations were measured using a liquid chromatography coupled to tandem mass spectrometry (LC–MS/MS) using 1 × 10^6^ cells for cell models and minimum 2 × 10^6^ cells for PBMCs, as described previously [5]. Briefly, cells were resuspended in 1 ml of isopropyl alcohol/water (80:20, v/v), 25 µl of the cell solution was mixed with 25 µl of internal standards (C16:0 Cer, C24:1 Cer, C16:0 GluCer, C24:1 GluCer, d18:1-S1P, d18:0 sphinganine, and d18:1 sphingosine). Separation of sphingolipids was performed by high-performance liquid chromatography (Shimadzu Scientific Instrument Inc., Columbia, MD, USA) and quantification using a 6500 LC–MS/MS system (AB Sciex, Concord, ON, Canada). Data analysis was done using the Analyst software version 1.7.2 (AB Sciex), as described in detail [5].

### Cell models and culture

Leukemic B cell models, JVM2 and HG3, were purchased from DSMZ (Braunschweig). Knockdown (KD) of *UGCG* was performed in HG3 and JVM2 cells by transduction with four validated lentiviral shRNA targeting the coding sequence of human *UGCG* (Sigma-Aldrich, Oakville, ON, Canada), as described in Supplementary Material and Methods. shUGCG2 and shUGCG4 (Supplementary Table 4) were used for the subsequent experiments as they displayed the highest efficiency to KD *UGCG* by mRNA level (> 50%). For treatment with sphingolipid inhibitors, UGCGi eliglustat and ibiglustat, SPHKi, fingolimod and SKI-II and ceramide synthases inhibitor (CERSi), fumonisin B1 were prepared in DMSO (Sigma-Aldrich) to obtain a working solution of 50 mM (for UGCGi and SPHKi) and 14 mM for CERSi. JVM2 and HG3 cells were plated at a density of 5 × 10^5^ and 1 × 10^5^ cells/mL, respectively, and treated for 72 h with various concentrations of sphingolipid inhibitors alone, or in combination with ibrutinib (1.8 µM and 3.6 µM). Concentrations of ibrutinib correspond to the IC_50_ and IC_50/2_ values determined in JVM2 cells [17]. Cell viability and apoptosis assays descriptions are provided in Supplementary Material and Methods.

### Drug synergy analysis

Drug synergy was assessed in CLL cell models using Combenefit (version 2.021) and SynergyFinder + (www.synergyfinderplus.org) softwares [18, 19]. The drug combination was quantified by comparing the obtained drug combination response in experimental data versus the expected response using the mathematical model Loewe additivity. Using Combenefit, a synergy score was calculated with a synergy score > 0 representing a synergistic response, an additive response if the score = 0, and antagonistic if the score < 0. With SynergyFinder + , a combination index (CI) was calculated with CI < 1 representing a synergy, CI = 1 an additive response, and > 1 an antagonistic combination.

### Statistical analysis

Comparison of data mean values between two groups was performed using a paired or unpaired two-tailed Student’s *t*-test. Prior to *t* tests, F-tests were performed to compare the variance between two groups of samples. A *P*-value < .05 was considered statistically significant.

## Results

### Inhibiting UGCG suppresses CLL viability and synergizes with ibrutinib

To mitigate the adverse effects of GluCer, two leukemic cell models were initially treated with UGCG inhibitors, eliglustat and ibiglustat (Supplementary Fig. 1). Treatment with each inhibitor resulted in a significant reduction in cell viability of JVM2 cells (Fig. 1A and B). The efficacy of UGCGi was confirmed through an assessment of intracellular sphingolipids using a targeted MS assay, with particular emphasis on measuring C16:0 and C24:1 Cer and GluCer. These specific sphingolipid species have been extensively quantified and observed to be altered in CLL according to prior research [5, 6] (Fig. 1C, D and Supplementary Fig. 2). The treatment with UGCGi led to a significant decrease in GluCer levels, with reductions of at least sevenfold for C16:0 GluCer (*P* < 0.001) and fourfold for C24:1 GluCer (*P* < 0.001) for both eliglustat and ibiglustat. As a result, there was an accumulation of Cer levels by more than fivefold (*P* < 0.05). These findings were consistent in HG3 cells, as shown in Supplementary Figs. 3 and 4.

In primary cells isolated from five untreated CLL patients whose characteristics are depicted in Supplementary Table 2, eliglustat treatments resulted in a significant reduction in GluCer levels (by twofold; *P* < 0.001 for 50-µM treatment) and Cer accumulation (by 1.5-fold; *P* < .001 for 50-µM treatment, Fig. 1E and Supplementary Fig. 5A). These treatments resulted in a significant decrease in cell viability (Fig. 1F) and an increase in cell death (Fig. 1G). Eliglustat significantly reduced cell viability by 22% compared to the vehicle (*P* = .02 for 50-µM treatment) and increased cell death by nearly 60% (*P* < .01, for 50-µM treatment). As the repression of GluCer resulted in the accumulation of Cer, we aimed to examine the effects of both sphingolipids on cell apoptosis and viability of primary CLL cells from 12 patients (Supplementary Table 3). We confirmed that the extracellular supplementation of C16:0 GluCer and C16:0 Cer led to a significant increase in intracellular sphingolipid levels of the corresponding sphingolipid (Fig. 1H and Supplementary Figs. 5B and 6). Cell viability was slightly reduced following C16:0 Cer treatment (Fig. 1I), although it did not reach significance. In contrast, C16:0 GluCer did not induce cell apoptosis and did not affect viability of primary CLL cells (Fig. 1I and J). In a recent report, it was demonstrated that C16:0 GluCer stimulates cell proliferation in actively dividing CLL cell models [5], a response not observed in primary CLL cells which exhibit limited proliferative activity. The finding that viability and apoptosis in primary CLL cells are unaltered by C16:0 GluCer aligns with its potential pro-proliferative impact.

**Fig. 2:**
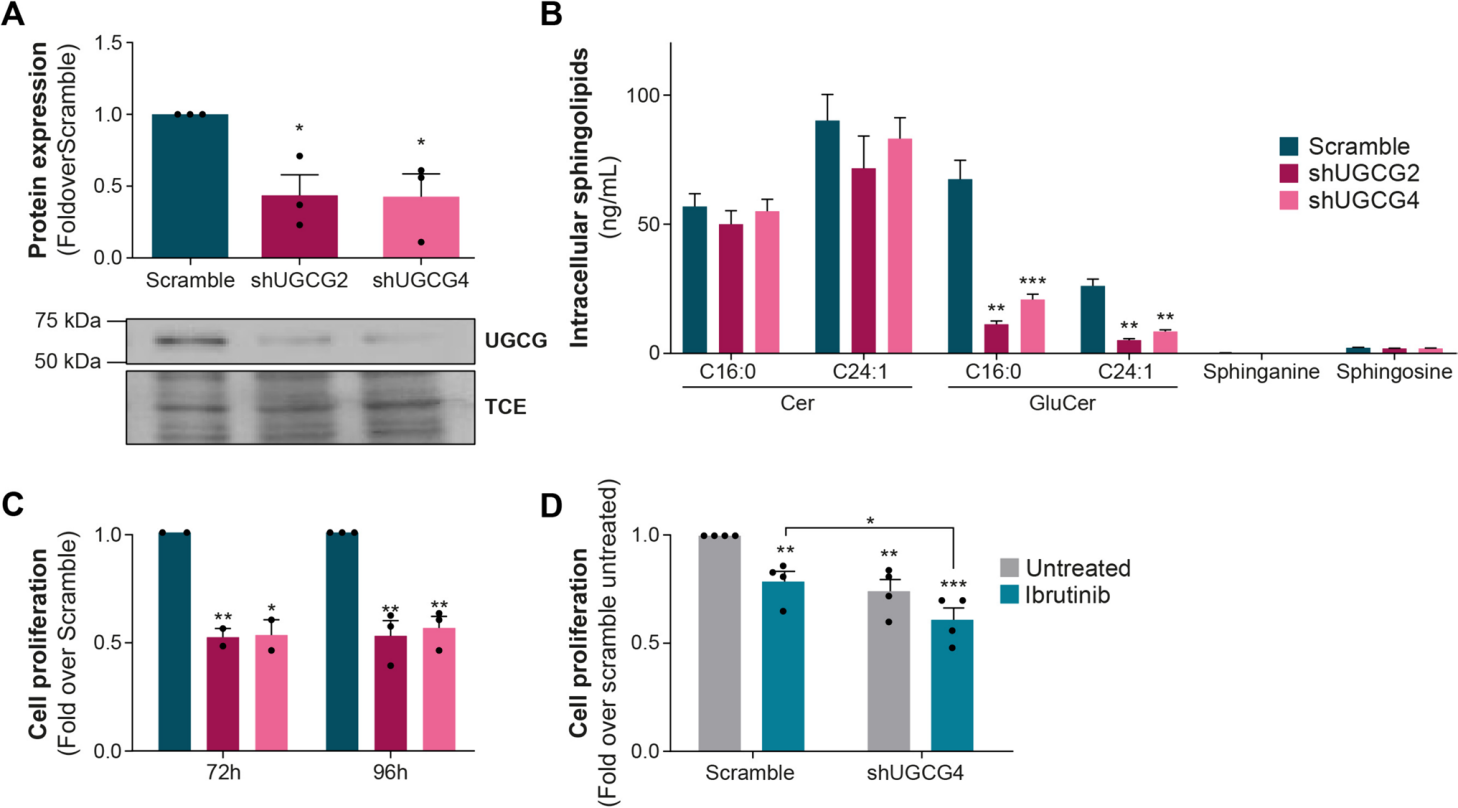
UGCG repression impairs leukemic cell proliferation. Knockdown (KD) of UGCG in JVM2 was validated at protein levels (**A**) and by measuring UGCG activity through quantification of intracellular sphingolipids (**B**). Data are expressed as the mean ± standard error from at least three independent experiments. (**C**) The effect of UGCG knockdown on cell proliferation was assessed by cell counting where cells were seeded at 0.1 × 10^6^ cells/mL in 5 mL, and viable cells were counted every 24 h for 4 days. Cell proliferation is calculated as fold over scramble. (D) Cells were treated with 0.3 µM ibrutinib for 96 h, and viable cells were counted for each condition. Cell proliferation is calculated as fold over untreated scramble. Data are expressed as the mean ± standard error from minimum two independent experiments. *P < .05; **P < .01; and ***P < .001. TCE: 2,2,2-trichloro-ethanol

**Fig. 3:**
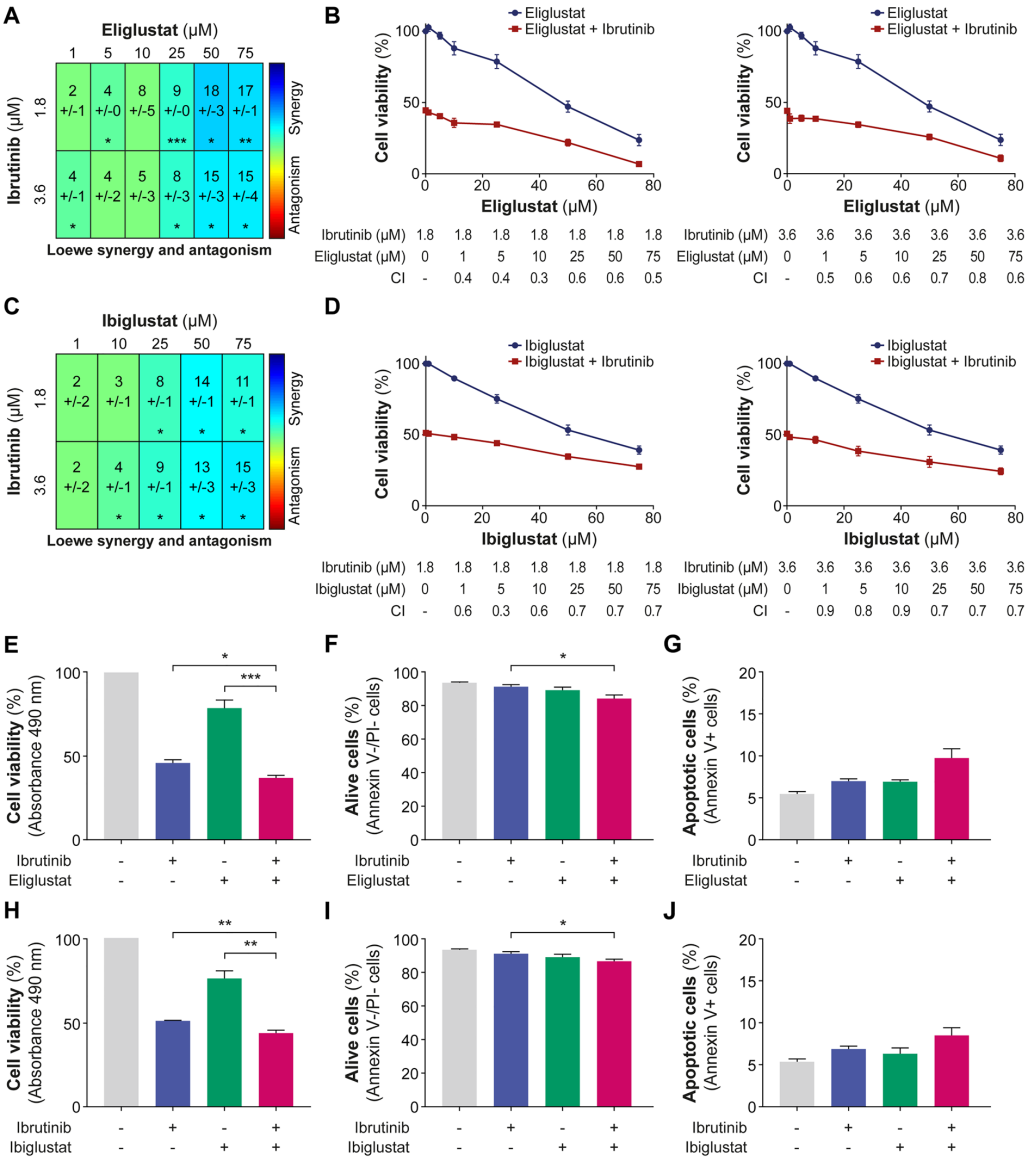
UGCG inhibition synergizes with ibrutinib in leukemic cells. JVM2 cells were co-treated for 72 h with IC_50_ or IC_50/2_ concentrations of ibrutinib (1.8 and 3.6 µM) as documented previously [17] along with increasing concentrations of UGCG inhibitors: eliglustat (**A**, **B**) and ibiglustat (**C**, **D**). Effect of the treatment combination was calculated using the Loewe additivity model using two softwares: SynergyFinder + and Combenefit. The synergy score is represented in a heatmap using Combenefit (**A**, **C**) and in graphs representing the combination index (CI) using SynergyFinder+ (**B**, **D**). With Combenefit, a synergy score > 0 represents a synergy, additive if the score = 0, and antagonistic if the score < 0. With SynergyFinder+, CI < 1 represents a synergy, CI = 1 is additive, and > 1 is antagonistic. (**E**–**J**) JVM2 cells were co-treated with 1.8 µM ibrutinib and 25 µM UGCGi for 72 h. (**E**, **H**) Cell viability was measured by MTS assay at 490 nm and calculated compared to untreated cells. Apoptosis and cell viability were assessed by Annexin V and propidium iodide (PI) staining using flow cytometry. JVM2 cells that were Annexin V-/PI- were considered alive (**F**, **I**) and Annexin V+ as apoptotic (**G**, **J**). *P*-values for the co-treatment were calculated against ibrutinib treatment alone and sphingolipid inhibitor treatment alone. Data are expressed as the mean ± standard error from minimum three independent experiments. *P < .05; **P < .01; and ***P < .001

**Fig. 4:**
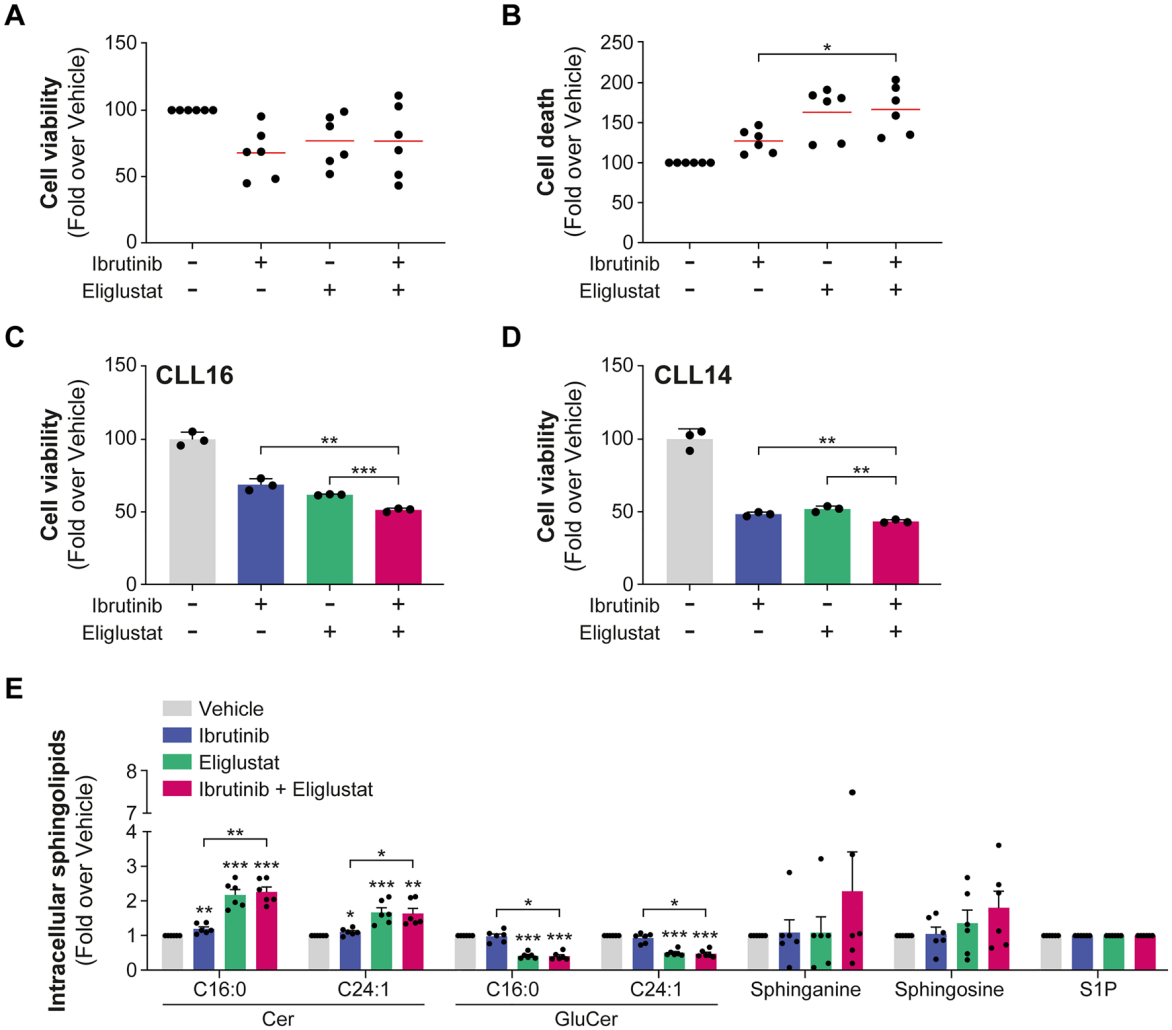
Combining UGCGi with ibrutinib in primary CLL cells decreased cell viability particularly in specific patients. (A-E) Primary cells from six CLL patients were treated with vehicle (DMSO), 0.3 µM ibrutinib, and 50 µM eliglustat alone and in combination for 46 h. (**A**, **B**) Cell viability was measured by CellTiter-Blue assay and cell death by FACS using DAPI-positive cells and calculated in fold over vehicle. Each dot representing one patient and the red line corresponding to mean values. (**C**, **D**) Cell viability measured in triplicate for selected patients who were sensitive to the drug combination is represented. Other four patients are represented in Supplementary Fig. 8. (**E**) Effects of ibrutinib, eliglustat, and the combination of both drugs on sphingolipid metabolism were assessed by mass spectrometry. Data are expressed as the mean ± standard error. *P < .05; **P < .01; and ***P < .001

To underscore the significance of targeting UGCG, we employed a shRNA method to repress *UGCG* expression in both JVM2 and HG3 cell lines, achieving significant reduction at the protein and enzyme activity levels (Fig. 2A, B and Supplementary Fig. 7A, B). For instance, in JVM2 cells, UGCG protein expression was repressed by 57% (*P* = 0.02, Fig. 2A) and C16:0 GluCer and C24:1 GluCer levels reduced by fivefold (*P* < .001, Fig. 2B), demonstrating a substantial decrease in UGCG activity. A similar outcome was observed with a second shRNA. Repression of UGCG significantly slowed cell proliferation by at least 40% (*P* < .05; Fig. 2C). These observations remained consistent within the HG3 cell line, despite a partial suppression of UGCG expression (Supplementary Fig. 7C). These observations strongly support the notion that UGCG activity and GluCer intracellular levels play a significant role in promoting the survival and proliferation of CLL cells. Leveraging data from the DepMap project, the impact of *UGCG* knockout (KO) by CRISPR/Cas9 on cell growth and survival could be assessed in various human cancer cell lines. *UGCG* KO was linked to diminished cell growth and survival across several types of human cancer cell lines (Supplementary Fig. 8A). Notably, among lymphoid cancer cell types, the top five cell lines most susceptible to *UGCG* KO included the CLL cell line MEC1 (Supplementary Fig. 8B). These findings further support the oncogenic role of UGCG expression and its enzymatic products GluCer, particularly in leukemic B cells, aligning with our previous prognostic observations in CLL patients [5]. In JVM2 cells with UGCG KD, ibrutinib led to a significant reduction in cell proliferation (by 20%; *P* < .05) compared to the control group (Fig. 2D), also confirmed in HG3 cells (Supplementary Fig. 7D). These results suggest that decreasing GluCer levels enhances the effectiveness of ibrutinib in inhibiting cell proliferation, highlighting the potential advantages of combining both approaches. Next, we investigated the potential of targeting UGCG to assess its effects when combined with anticancer agents such as the targeted agent ibrutinib. When combining UGCGi with ibrutinib, a synergistic effect was observed in both JVM2 (Fig. 3A–D) and HG3 (Supplementary Fig. 9) cell models. This was supported by a reduced cell viability by over 50% using an MTS assay (Fig. 3E). No notable effect on cell death and apoptosis was noted by flow cytometry (Fig. 3F and G). Similar observations were noted for ibiglustat (Fig. 3H–J) suggesting that the combination of UGCGi and ibrutinib may not impact cell death but affects cell proliferation supporting earlier observations. The combination of eliglustat with ibrutinib showed an impact on primary CLL cell survival that did not reach significance compared to eliglustat treatment alone when all patients were considered (Fig. 4A, B and Supplementary Table 2). However, we noted significant interindividual variability, where CLL cells from certain patients exhibited greater sensitivity to the combination of eliglustat with ibrutinib, resulting in a significant decrease in cell viability compared to either treatment alone (Fig. 4C, D and Supplementary Fig. 10). This drug sensitivity could suggest a higher proliferative capacity of CLL cells from these patients compared to those in whom the inhibitor has no effect. Interestingly, responders were all young women and exhibited aggressive disease characteristics, such as high CD38 expression and absence of 13q deletion (Supplementary Table 1). Furthermore, the drug combination resulted in a significant decrease in GluCer levels (by twofold, *P* < .001, Fig. 4E) and accumulation of Cer levels by 1.6-fold (*P* < .05) compared to untreated and ibrutinib treatment conditions but no significant changes compared to eliglustat treatment. This observation suggests that the combination of eliglustat and ibrutinib impact on sphingolipid metabolism is only induced by eliglustat.

### Exploring the targeting of alternative enzymes of the sphingolipid pathway in CLL

**Fig. 5:**
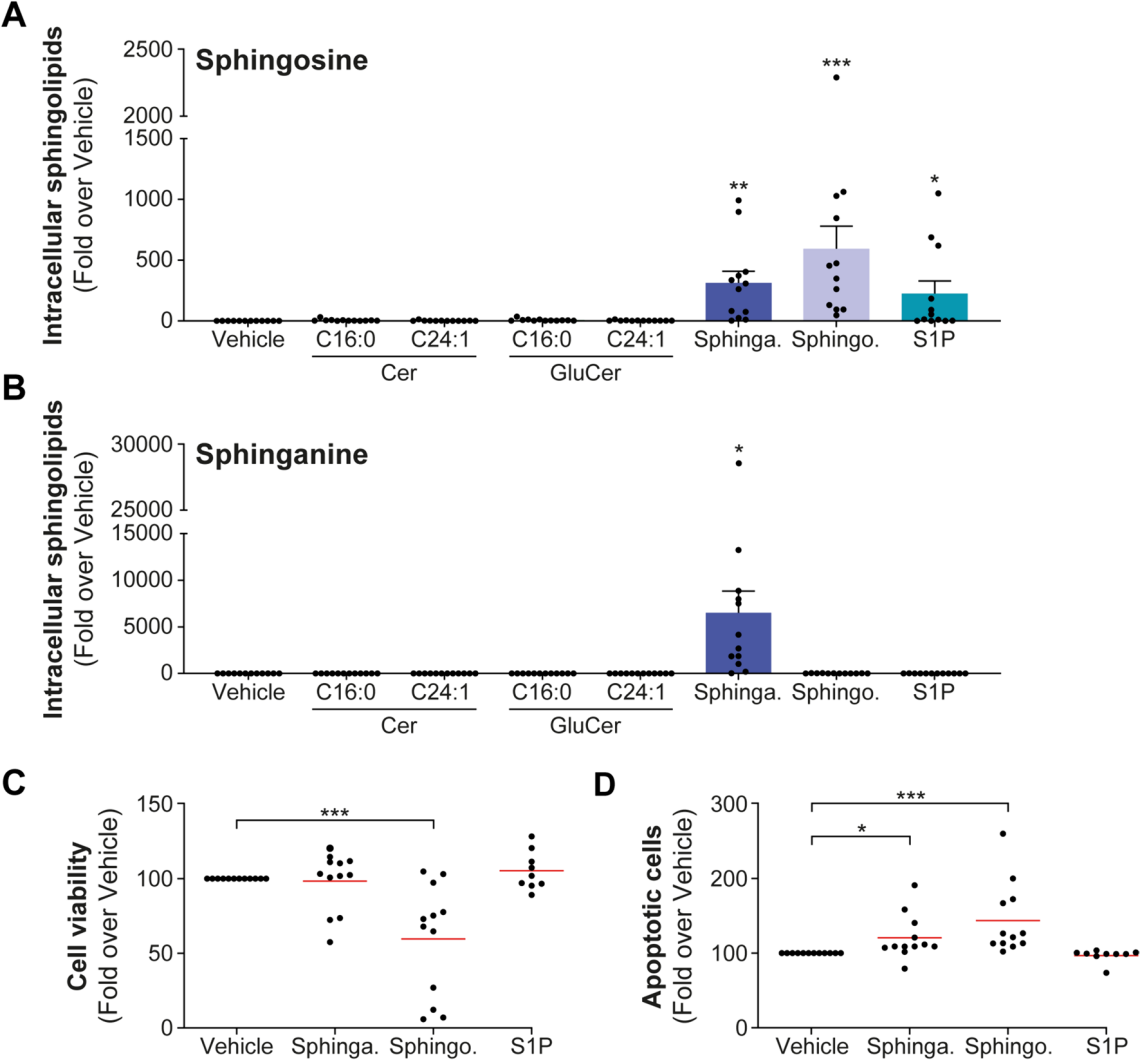
Treatments with sphingosine and sphinganine impact the survival of primary CLL cells. (**A**–**D**) Primary cells from 12 CLL patients were treated with 10 µM sphingosine, sphinganine, S1P, or vehicle (MeOH) for 22 h. Efficacy of sphingosine (**A**) and sphinganine (**B**) treatments was assessed by targeted lipidomics and confirmed accumulation of intracellular sphingolipids. Cell viability was assessed by CellTiter-Blue assay (**C**) and apoptosis (**D**) by FACS using DAPI and Annexin V stainings and calculated compared to vehicle. Red lines represent the mean values. Data for sphingolipids quantification are expressed as the mean ± standard error. *P < .05; **P < .01; and ***P < .001. Sphinga.: sphinganine; sphingo.: sphingosine; and S1P: sphingosine-1-phosphate

**Fig. 6:**
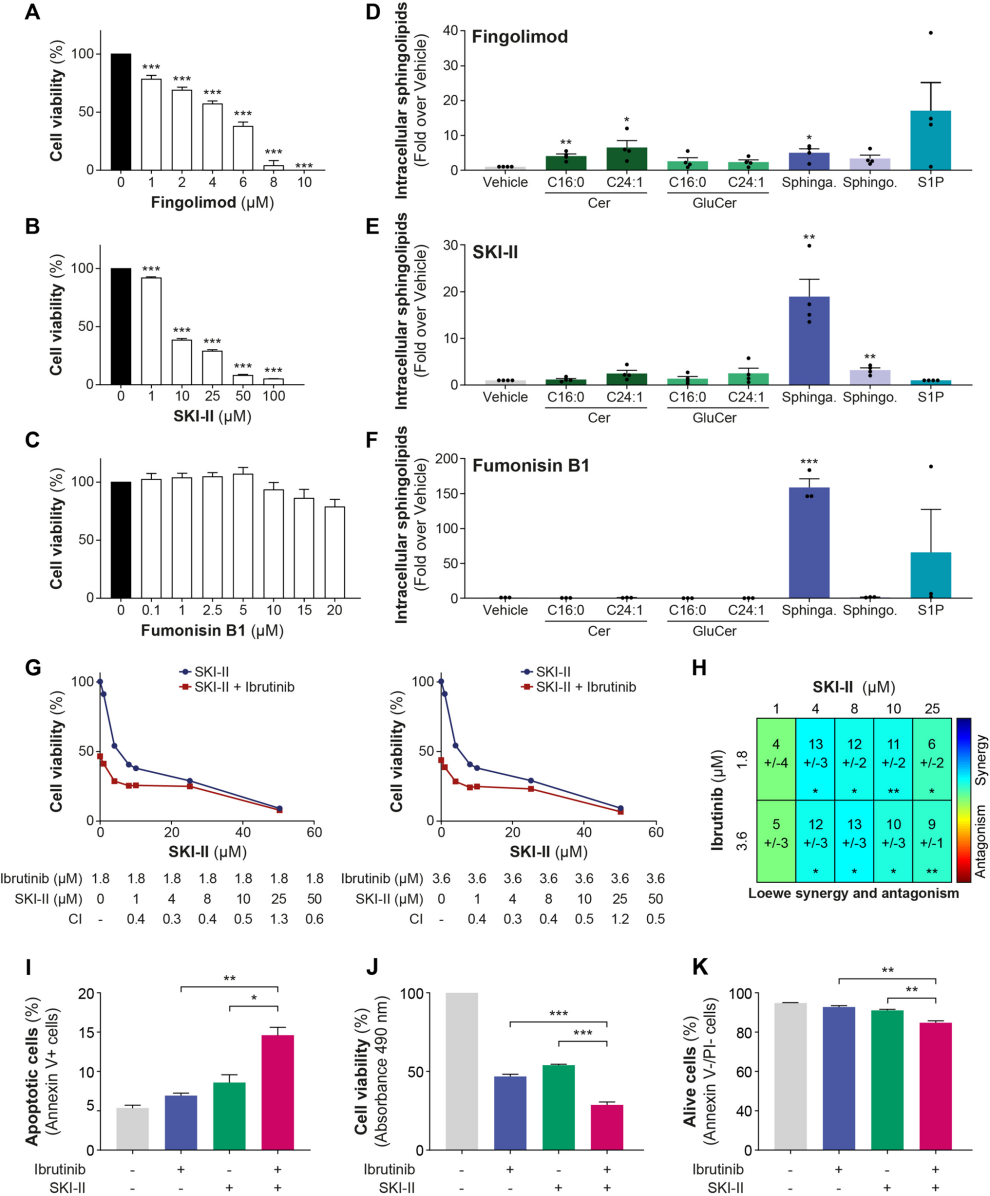
SPHKi reduces cell viability in leukemic B cells and primary CLL cells and synergizes with ibrutinib. JVM2 cells were treated with increasing concentrations of SPHK inhibitors fingolimod (**A**), SKI-II (**B**), and CERS inhibitor, fumonisin B1 (**C**) for 72 h, cell viability was measured by MTS assay at 490 nm and calculated compared to untreated cells. (**D**–**F**) Efficacy and specificity of the inhibitors were assessed by quantifying intracellular sphingolipids concentration. JVM2 cells were treated with IC_50_ concentrations of SPHKi for 72 h corresponding to 2 µM fingolimod (**D**) and 8 µM SKI-II (**E**) and 20 µM fumonisin B1 (**F**). (G-H) JVM2 cells were co-treated for 72 h with IC_50_ or IC_50/2_ concentrations of ibrutinib (1.8 and 3.6 µM) and increasing concentrations of SKI-II. Effect of the treatment combination was calculated using the Loewe additivity model. The synergy was represented in graphs representing the combination index (CI) using SynergyFinder + software (**G**) and in a heatmap using Combenefit software (**H**). With SynergyFinder + , CI < 1 represents a synergy, CI = 1 is additive, and > 1 is antagonistic. With Combenefit, a synergy score > 0 represents a synergy, additive if the score = 0, and antagonistic if the score < 0. (I-K) JVM2 cells were co-treated with 1.8 µM ibrutinib and 4 µM SKI-II for 72 h. Apoptosis was assessed by Annexin V and propidium iodide (PI) stainings using flow cytometry. Cells that were Annexin V + were considered apoptotic (**I**) and Annexin V-/PI-, alive (**J**). (**K**) Cell viability was measured by MTS assay at 490 nm and calculated compared to untreated cells. *P*-values for the co-treatment were calculated against ibrutinib treatment alone and SKI-II treatment alone. Data are expressed as the mean ± standard error from three independent experiments. *P < .05; **P < .01; and ***P < .001. Sphinga.: sphinganine; sphingo.: sphingosine; and S1P: sphingosine-1-phosphate

To determine whether other enzymes involved in the metabolism of sphingolipids could be potential targets in CLL, inhibitors targeting sphingosine kinases, SKI-II and sphingosine kinase 1, fingolimod, along with the ceramide synthase inhibitor (CERS) fumonisin B1 were evaluated (Supplementary Fig. 1). These investigations are primarily based on the association between circulating sphinganine levels and patient survival [5]. Initially, we assessed the effect of sphingolipids in primary cells from 12 CLL patients to affect cell viability and apoptosis. Sphingolipid treatments led to significant intracellular accumulations by over 500-fold (*P* < .01, Fig. 5A) for sphingosine and an even greater accumulation of both sphinganine (*P* < .05, Fig. 5B) and S1P (*P* < .10, Supplementary Fig. 11). Intracellular accumulation of sphingosine significantly reduced viability of primary CLL cells by 40% (*P* = .001, Fig. 5C). In addition, both sphinganine- and sphingosine-induced apoptosis (by 21%; *P* = .03 and 44%; *P* = .004, respectively), with no effect observed for S1P (Fig. 5D). These findings suggest that an elevated intracellular concentration of sphinganine and sphingosine triggers cell death in primary CLL cells but not S1P, implying their potential as therapeutic targets in CLL. This notion was examined initially in cell models. Fingolimod and SKI-II significantly reduced cell viability in a dose-dependent manner in JVM2 cells (Fig. 6A and B). In contrast, fumonisin B1 did not affect cell viability (Fig. 6C). Assessment of intracellular sphingolipids confirmed the effectiveness of these treatments (Fig. 6D–F). Fingolimod led to a significant increase in sphinganine and Cer by 4–6.5-fold (*P* < .05) and with no significant effect on S1P and sphingosine levels (Fig. 6D). SKI-II showed a more specific effects on the accumulation of sphinganine by 19-fold (*P* = .003) and more modestly sphingosine by threefold (*P* = .004), respectively (Fig. 6E). Fumonisin B1 was more efficient at inducing an accumulation of sphinganine (159-fold; *P* < .001) (Fig. 6F). These observations were further confirmed in HG3 cells (Supplementary Fig. 12A-F). Since fumonisin B1 did not impact cell viability, and fingolimod's effect on sphingolipid metabolism was not as specific as SKI-II, subsequent experiments were pursued using the SKI-II inhibitor.

**Fig. 7:**
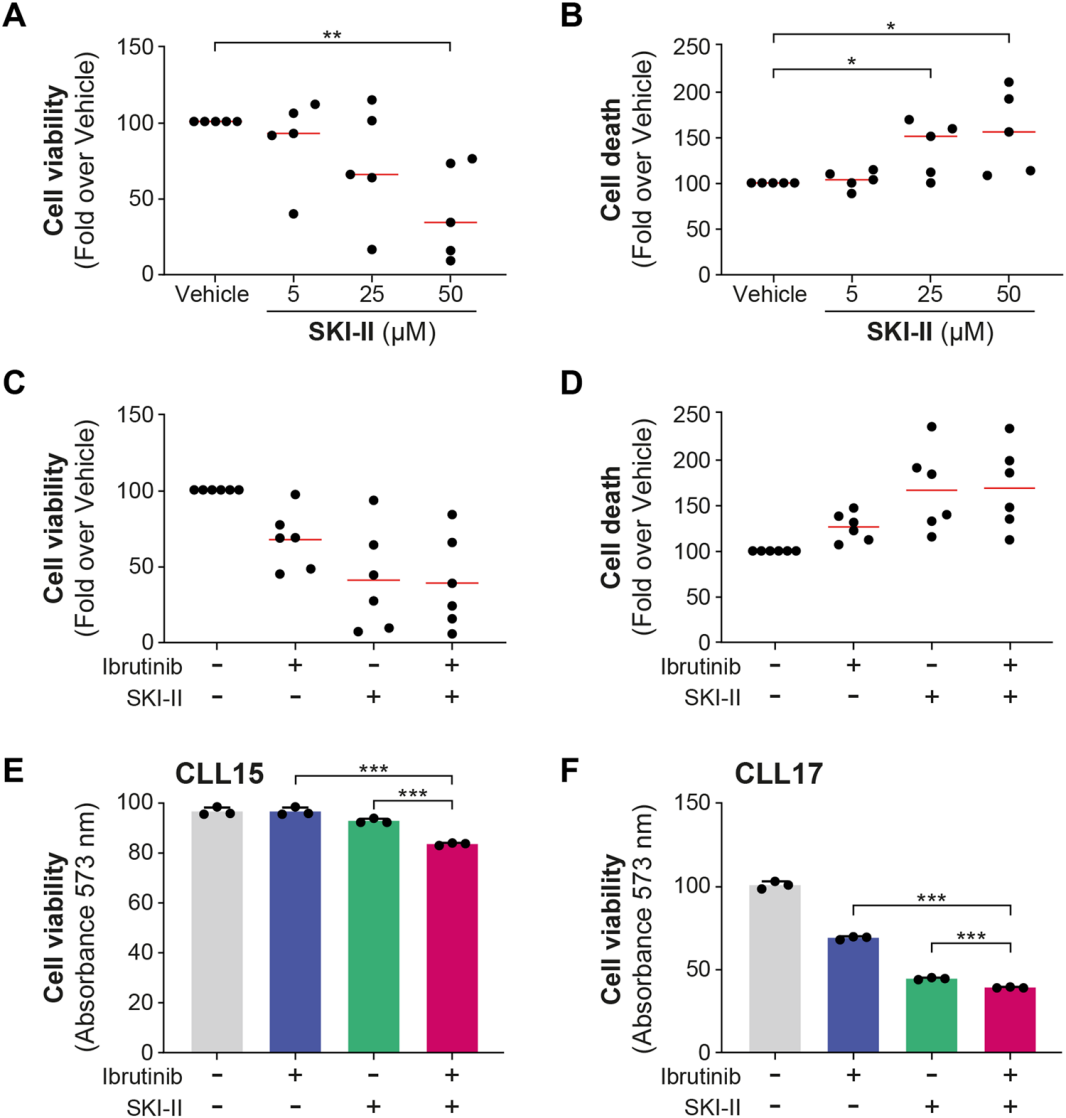
SPHKi alone and in combination with ibrutinib reduces cell viability in primary CLL cells, particularly in specific patients. (**A**, **B**) Peripheral blood mononuclear cells (PBMCs) from five CLL patients were treated with 5, 25, and 50 µM SKI-II for 46 h. Cell viability was measured by CellTiter-Blue assay and cell death by FACS using DAPI-positive cells and calculated in fold over vehicle. (**C**–**F**) PBMCs from six patients were treated with vehicle (DMSO), 0.3 µM ibrutinib, and 50 µM SKI-II alone and in combination for 46 h. (**C**, **D**) Cell viability and cell death were calculated in fold over vehicle and were represented in graph in which one dot corresponds to one patient. (**E, F**) Cell viability measured in triplicate for selected patients was represented. Red line corresponds to the mean values. Data are expressed as the mean ± standard error. *P < .05; **P < .01; and ***P < .001. S1P: sphingosine-1-phosphate

The combination of SKI-II with ibrutinib led to a synergistic effect in JVM2 (Fig. 6G–H), also confirmed in the second HG3 model (Supplementary Fig. 12G–H). The combination of SKI-II and ibrutinib at the concentration demonstrating the highest synergy markedly elevated apoptosis in JVM2 cells compared to individual treatments (*P* < .05, Fig. 6I). The co-treatment notably decreased the percentage of viable cells (*P* < .01, Fig. 6K), further confirmed by MTS assay results (*P* < .001, Fig. 6J), indicating that the combination of SKI-II and ibrutinib might affect both apoptosis and cell proliferation in JVM2 cells. In primary cells from five CLL patients, SKI-II negatively impacted viability in a dose-dependent manner (Fig. 7A and B). SKI-II treatments primarily increased sphinganine levels in primary cells as observed in cell models, although it did not reach significance. Significant increase in C16:0 Cer was noted following SKI-II treatments, which could be caused by an elevation of sphingosine bioconversion to Cer (Supplementary Fig. 13). Using 50 µM of SKI-II, cell viability was reduced by 58% (*P* = .003) and cell death enhanced by 56% (*P* = .025). The combination of SKI-II with ibrutinib did not significantly affect CLL cell survival for all patients compared to either treatment alone (Fig. 7C and D). However, as observed for UGCGi, CLL cells from a subset of patients exhibited greater sensitivity to the combination of SKI-II with ibrutinib, with a significant decrease in cell viability compared to either treatment alone (Fig. 7E and F), potentially supporting the relevance of targeting this pathway.

The combination of both eliglustat and SKI-II was evaluated in JVM2 and HG3 cell models based on the observation above and the distinct associations of GluCer and sphinganine accumulation with survival [5]. Combination of eliglustat and SKI-II resulted in a synergistic effect on both cell lines (Supplementary Fig. 14), supporting the potential of using both sphingolipid pathway inhibitors in CLL treatment.

## Discussion

**Fig. 8:**
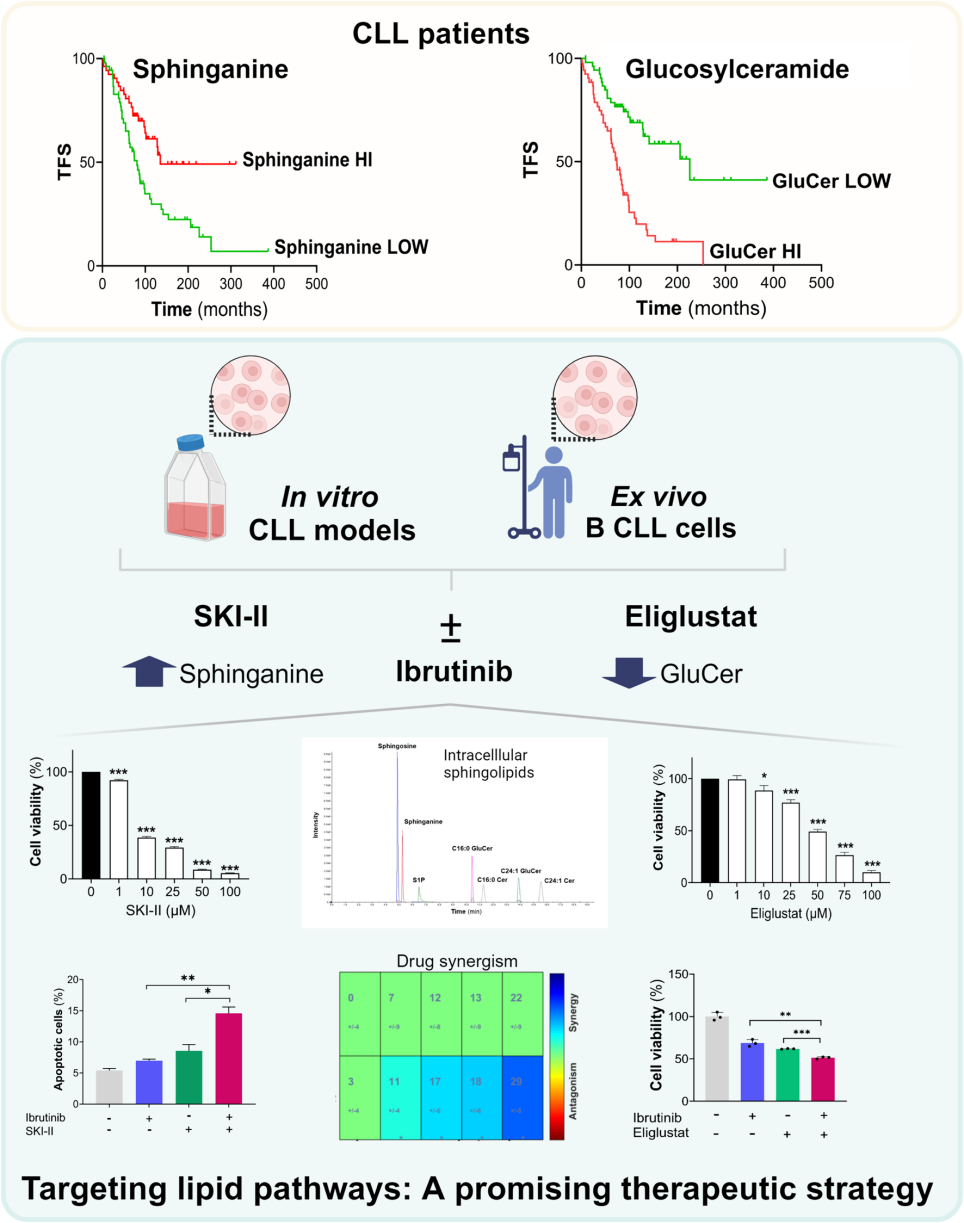
Schematic summary representation of the research. High circulating levels of glucosylceramide (GluCer) were significantly associated with poor treatment-free survival (TFS) of CLL patients. Inversely, higher circulating levels of sphinganine were associated with prolonged TFS. Inhibitors targeting the sphingolipid metabolism pathway were evaluated using ex vivo and in vitro models, showing significant reductions in cell viability. Eliglustat inhibits the UDP-glucose ceramide glucosyltransferase leading to decreased intracellular levels of GluCer and SKI-II targets sphingosine kinases and delta-4 desaturase sphingolipid enzymes resulting in increased levels of sphinganine measured by targeted mass spectrometry. Additionally, they exhibited a synergistic effect when combined with ibrutinib. This study highlighted a new potential therapeutic avenue in CLL by targeting the sphingolipid metabolism pathway. Created with BioRender.com

Findings support that inhibiting UGCG either pharmacologically or genetically has a detrimental impact on leukemic cells, providing further support of the pro-oncogenic potential of UGCG and GluCer in the context of CLL (Fig. 8). This aligns with observations from our previous study involving cohorts of CLL patients revealing the prognostic value of an accumulation in circulation of the UGCG products GluCer and elevated *UGCG* mRNA leukemic expression, both as adverse prognostic markers [5]. The detrimental effects of UGCGi on leukemic cells survival such as the approved eliglustat and its synergistic effect with ibrutinib suggest that incorporating UGCGi could enhance the benefits of ibrutinib treatment. We further demonstrate that targeting oncogenic SPHK lipid kinases by impacting the accumulation of other sphingolipids could represent another promising therapeutic avenue in CLL.

Targeting UGCG has demonstrated efficacy in treating rare genetic disorders such as Gaucher disease [20, 21], for which eliglustat has been approved, and Fabry disease, for which ibiglustat is currently under clinical evaluation (NCT02228460 and NCT2489344). Additionally, UGCG inhibition is being explored as a potential treatment for Parkinson's disease (NCT02906020) and kidney diseases in ongoing clinical trials (NCT03687554 and NCT04705051). However, research in cancer remains relatively unexplored. One of the primary reasons for the limited clinical application of UGCG inhibitors is likely the lack of understanding regarding the molecular actions of GluCer [22, 23]. UGCG inhibitors were shown to reduce tumor volume and tumor size in vivo models of renal cancer and melanoma [24, 25], respectively, and decreased proliferation of leukemic [5], prostate [26], breast [27], and glioma cancer cells [28]. As documented in this study, the dependency of cancer cells on the UCGC pathway was observed in other cancer cell lines, including some lymphoid cells such as KMS20 or MEC1 cells, which highlights the potential benefit of targeting GluCer production as a therapeutic approach in CLL.

In the context of CLL, very few studies have explored the use of UGCG inhibitors. In this study, changes in sphingolipid intracellular concentrations were also carefully monitored using a sensitive mass spectrometry assay confirming the efficacy of different treatment conditions. Our data support the notion that UGCG inhibitors efficiently lead to an accumulation of GluCer and that they can confer benefits both alone and in combination with ibrutinib, as evidenced by the decreased viability of cell models and primary CLL patients' cells. The observation that viability and apoptosis remain unchanged in malignant primary CLL cells following exposure to GluCer aligns with its previously observed pro-proliferative effect in CLL cell models [5], reinforcing the consistency of these findings. No previous studies reported the efficacy of inhibiting UGCG in reducing leukemic CLL cell viability, which could be attributed to the utilization of less specific UGCG inhibitors, such as imino sugars-based molecules [6, 14, 20] and inadequate alteration in intracellular GluCer levels [6]. Additional studies in CLL demonstrated that inhibiting UGCG sensitizes cells to various drugs such as fludarabine, chlorambucil, and inhibitor of BCL-2 (ABT-737) [6, 13, 14]. When evaluating the effects of UGCG inhibition on leukemic cells, distinguishing whether the observed effects stem from reduced GluCer levels or accumulation of Cer levels can pose a challenge [6, 29]. Ceramides were reported to induce cell death in various cell types [1], including leukemic cells [30–32]. However, in primary CLL cells, we observed no effects of Cer. In contrast, a prosurvival effect of GluCer with no detectable impact on apoptosis was seen in primary leukemic cells, further supporting our previous demonstration that GluCer levels are associated with CLL aggressiveness, rapid progression in CLL patients and pro-proliferative effects in the same leukemic cell models examined here [5]. This was corroborated by an elevation of various markers associated with protein synthesis, cell cycle regulation, and cell proliferation pathways following GluCer treatment [5]. We cannot exclude that the absence of Cer effects on CLL cell phenotype could be due to the hydrophobic features of these molecules, which result in poor internalization and lower intracellular accumulation compared to other sphingolipids [33]. A potential limitation for the clinical application of UGCG inhibitors might be their low bioavailability that can be partially addressed by combining them with anticancer treatments as reported in several studies [34, 35] and supported by our findings with combination of UGCG and ibrutinib. More recently, the remarkable in vivo anti-tumor effectiveness and tolerability demonstrated [36] also using the FDA-approved highly selective UGCG inhibitor eliglustat, underscores its clinical promise.

Targeting sphingosine kinases using SKI-II could represent another promising therapeutic option in CLL. SKI-II inhibitors were shown to decrease cell viability [9, 10]. SKI-II has been demonstrated to inhibit not only sphingosine kinases primarily regulating the balance of Cer and S1P, but also delta-4 desaturase, sphingolipid involved in the de novo synthesis pathway preventing the conversion of dihydroceramide to ceramide (Supplementary Fig. 1) [37]. Although dihydroceramide was not measured, this inhibition likely affects the accumulation of intracellular sphinganine, the precursor of dihydroceramide, as observed in cell models and primary B cells. This observation also aligns with our previous work, which emphasized that CLL patients with higher circulating levels of sphinganine had an improved treatment-free survival [5]. Mechanistically, increased sphinganine concentration in leukemic cells was found to induce apoptosis in vitro, leading to an upregulation of pro‐apoptotic factors [5]. Here, in primary cells of CLL patients, we further demonstrated the synergistic effect of SKI-II in combination with ibrutinib. A previous study by Almejun and colleagues highlighted that SKI-II enhances the effect of ibrutinib [10], which supports our findings suggesting that inhibiting SPHK could be another effective combinatorial approach with ibrutinib.

In summary, we propose targeting sphingolipid metabolism, particularly the UGCG pathway, as a promising new candidate for novel therapy in CLL. This approach could be further enhanced through combination with ibrutinib, as supported by findings in leukemic B cells and primary cells from CLL patients. Our results underscore the need for validation, which should involve a larger cohort of CLL patients and in vivo studies. This is essential to fully substantiate the potential therapeutic avenue identified in CLL.

## Supplementary Information

Below is the link to the electronic supplementary material.

Supplementary file1 (PDF 581 KB)

Supplementary file2 (PDF 255 KB)

## Data Availability

No datasets were generated or analyzed during the current study.
