# Supplementary material for: Targeting sphingolipid metabolism in chronic lymphocytic leukemia
Source: Clin Exp Med. 2024 Jul 30;24(1):174. doi: 10.1007/s10238-024-01440-x (PMC11289351; doi:10.1007/s10238-024-01440-x)
Supplement: Supplementary file 1 — Supplementary file1 (PDF 581 KB) [file 10238_2024_1440_MOESM1_ESM.pdf]

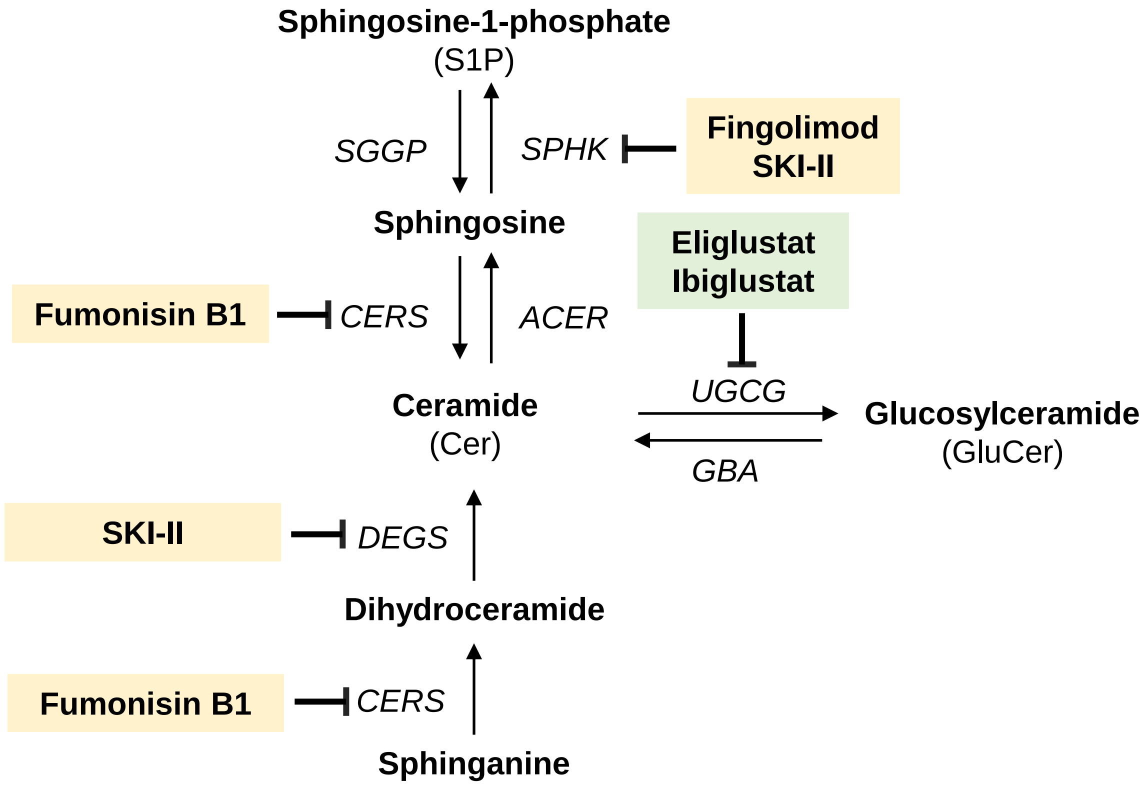

Supplementary Figure 1

A simplified diagram illustrating the sphingolipid biosynthetic pathway, along with the key targets of sphingolipid inhibitors.

Eliglustat and ibiglustat are inhibitors of UDP-glucose ceramide glucosyltransferase (UGCGi), the enzyme responsible for converting ceramide to glucosylceramide. Inhibition of UGCGi leads to a decrease in glucosylceramide production, favoring the accumulation of ceramides. Fingolimod and SKI-II are inhibitors of sphingosine kinases (SPHKi), enzymes involved in converting sphingosine to sphingosine-1-phosphate (S1P). Inhibition of SPHK results in decreased S1P production, leading to an accumulation of sphingosine. SKI-II also inhibits the the delta 4-desaturase sphingolipid (DEGS), responsible for converting dihydroceramide to ceramides, favoring an accumulation of dihydroceramide. Fumonisin B1 is an inhibitor of ceramide synthases (CERSi), the enzymes responsible for producing ceramides. Treatment with fumonisin B1 reduces ceramide production, favoring increased levels of sphinganine and sphingosine.

SGGP: sphingosine-1-phosphatase phosphatase; alkaline ceramidase; GBA: glucosylceramidase beta.

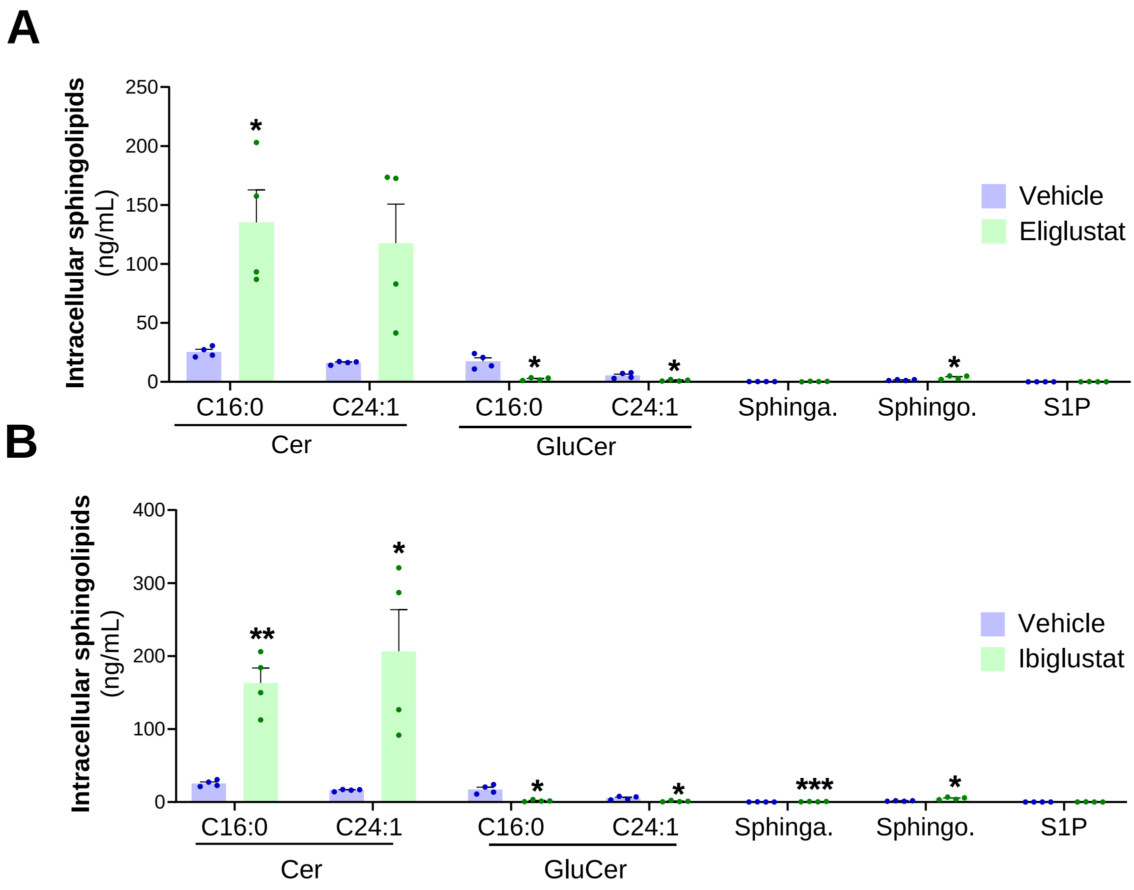

Supplementary Figure 2

(A-B) JVM2 cells were treated with IC_{50} concentrations of UGCG inhibitors (UGCGi) for 72h, 42 µM eliglustat (A) or 48 µM ibiglustat (B). Absolute quantification of intracellular sphingolipids was measured using a targeted lipidomic approach. Ibiglustat induces a significant increase of sphinganine by 3-fold (*P* = 0.007). Data are expressed as the mean ± standard error from four independent experiments. **P* < .05; ***P* < .01; ****P* < .001. Sphinga.: sphingosine; sphingo.: sphingosine; S1P: sphingosine-1-phosphate.

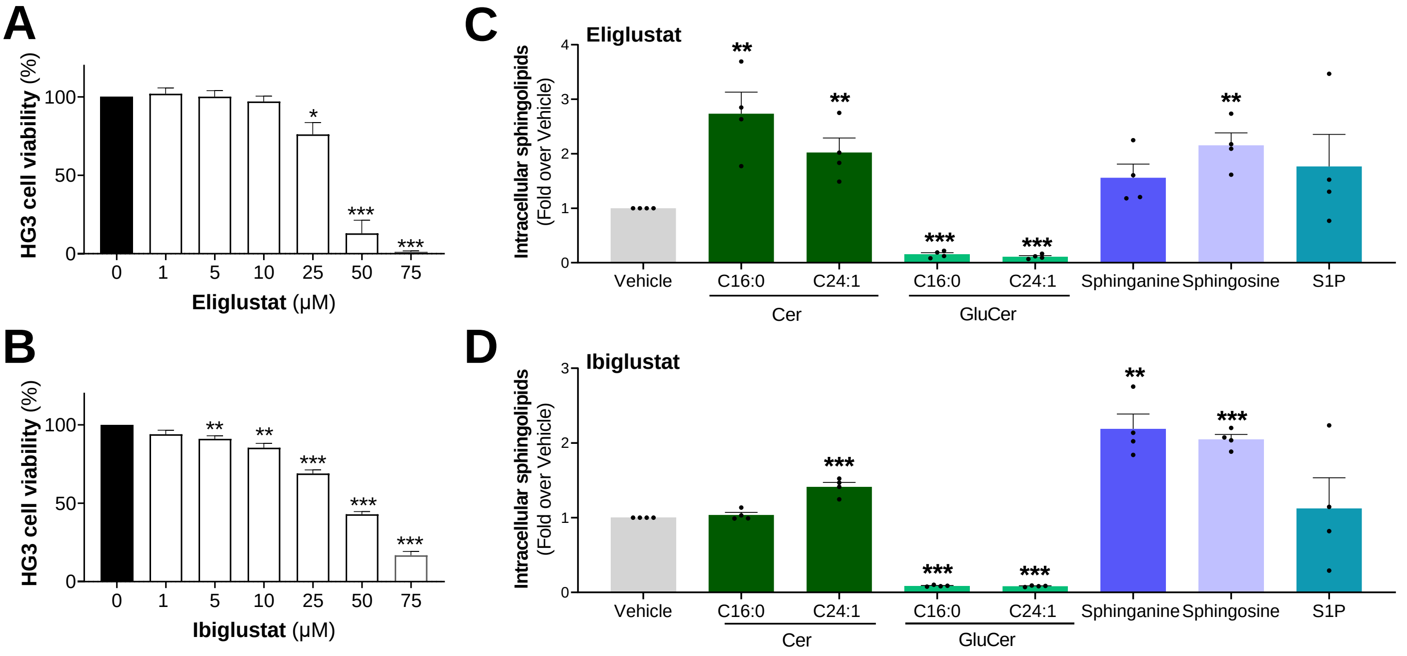

Supplementary Figure 3

HG3 cells were treated with UGCG inhibitors (UGCGi), eliglustat (A,C) or ibiglustat (B,D) for 72h. (A-B) Effect of UGCGi on cell viability was measured by MTS assay at 490 nm and calculated compared to untreated cells. (C-D) Efficacy and specificity of UGCGi were assessed by quantifying intracellular sphingolipids concentration. HG3 cells were treated with UGCGi IC_{50} concentrations for 72h corresponding to 30 µM eliglustat (C) and 37 µM ibiglustat. Data are expressed as the mean ± standard error from minimum two independent experiments. *P < .05; **P < .01; ***P < .001. Sphinga.: sphingosine; sphingo.: sphingosine; S1P: sphingosine-1-phosphate.

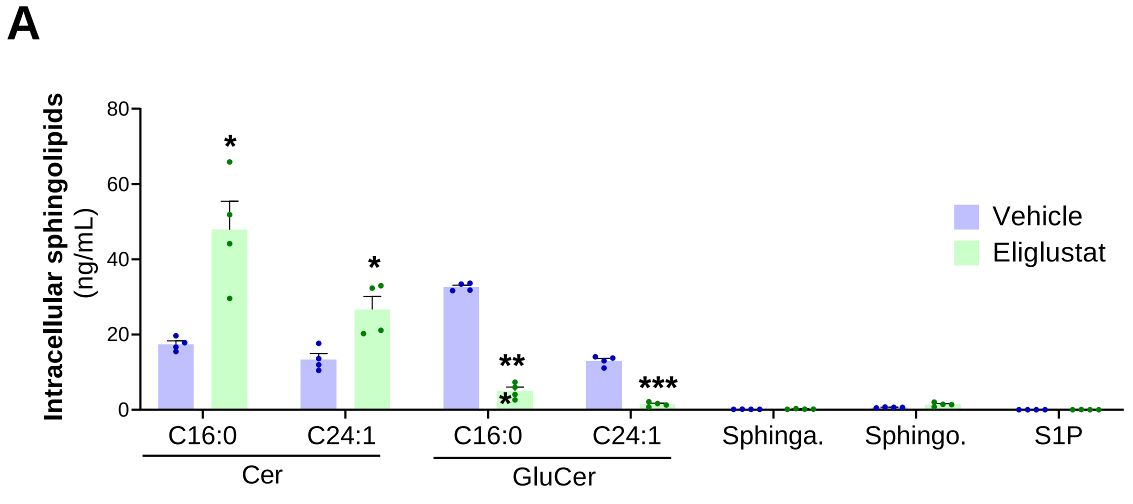

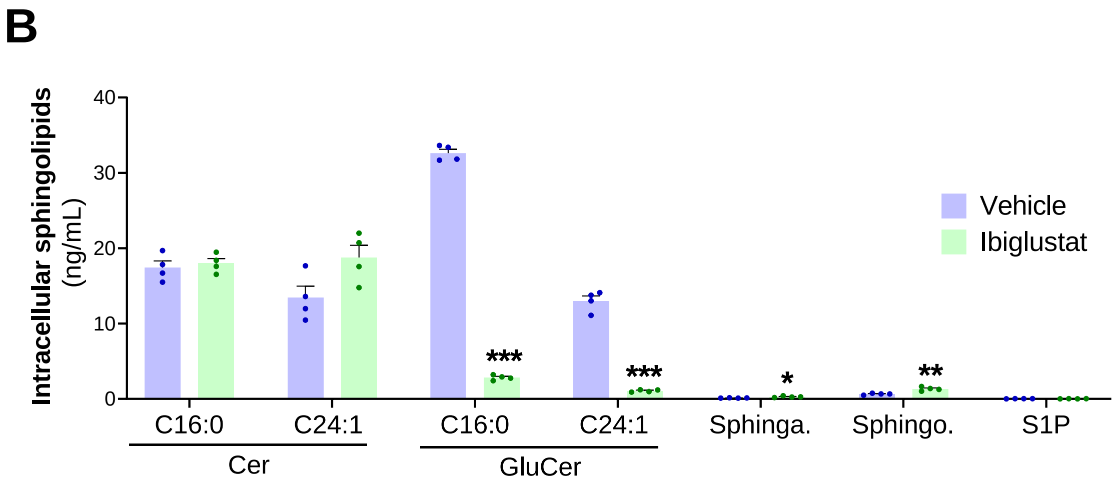

Supplementary Figure 4

(A-B) HG3 cells were treated with IC_{50} concentrations of UGCG inhibitors (UGCGi) for 72h, 30 µM eliglustat (A) or 37 µM ibiglustat (B). Absolute quantification of intracellular sphingolipids was measured using a targeted lipidomic approach. Ibiglustat induces a significant increase of sphinganine by 2-fold (P < 0.001). Data are expressed as the mean ± standard error from four independent experiments. *P < .05; **P < .01; ***P < .001. Sphinga.: sphingosine; sphingo.: sphingosine; S1P: sphingosine-1-phosphate.

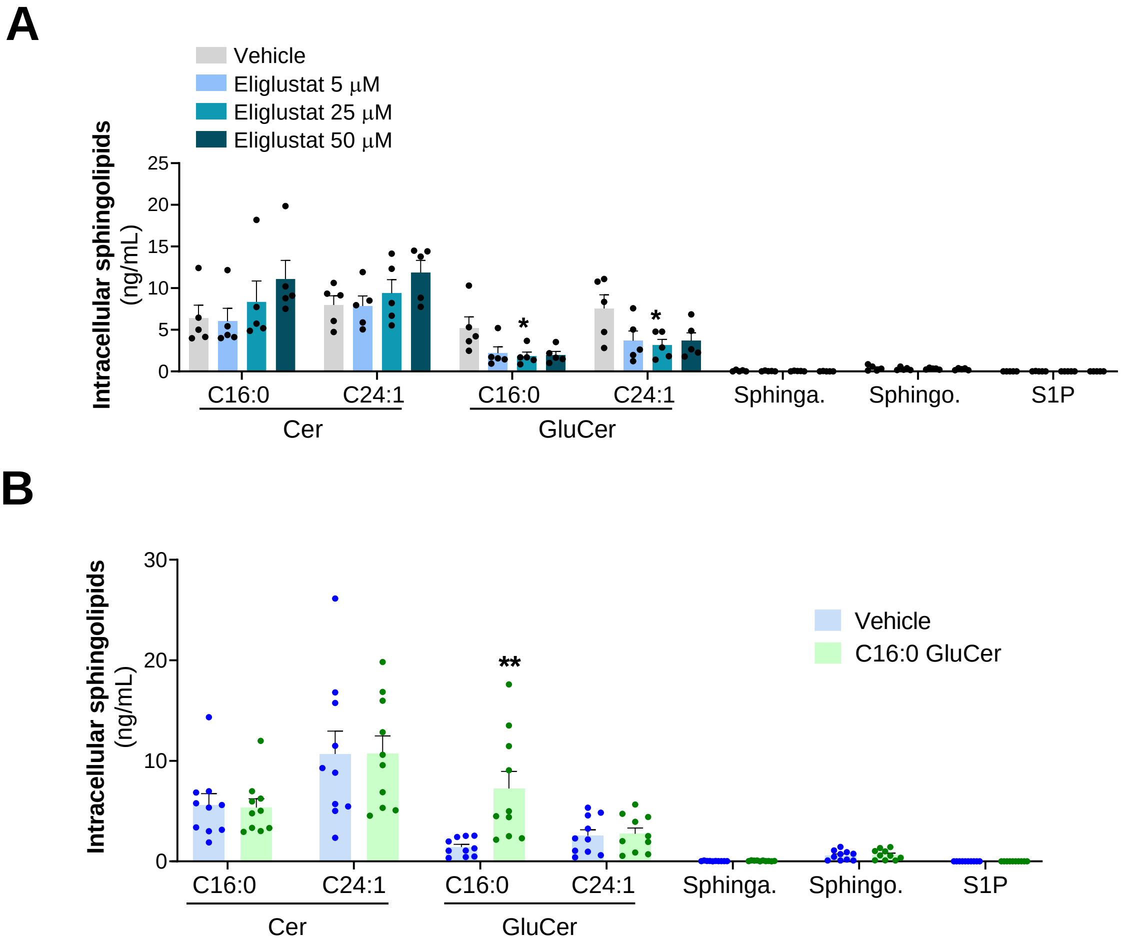

Supplementary Figure 5

(A) Peripheral blood mononuclear cells (PBMCs) from 5 CLL patients were treated with 5, 25 and 50 µM eliglustat for 46h, treatment efficiency was assessed by absolute quantification of intracellular sphingolipids by mass spectrometry (MS). (B) 12 primary cells from CLL patients were treated with 10 µM C16:0 GluCer (1:1 $CHCL_3$/MeOH) or vehicle for 22h. 10 out of 12 patients presented enhanced intracellular concentration of C16:0 GluCer confirmed by MS *P < .05; **P < .01; ***P < .001. Sphinga.: sphingosine; sphingo.: sphingosine; S1P: sphingosine-1-phosphate.

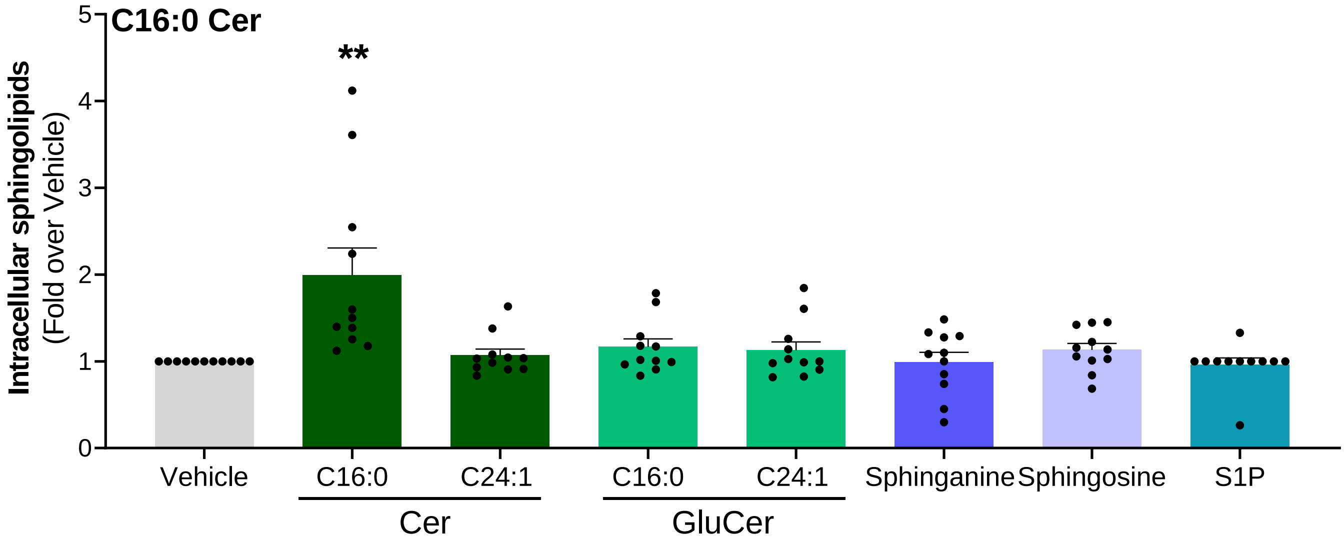

Supplementary Figure 6

Primary cells from 12 CLL patients were treated with 10 µM C16:0 Cer for 22h. Overall, 11 CLL patients out of 12 presented an accumulation of intracellular C16:0 Cer (1:1 $CHCL_3$/MeOH) confirmed by mass spectrometry. Data are expressed as the mean ± standard. **P* < .05; ***P* < .01; ****P* < .001. S1P : sphingosine-1-phosphate.

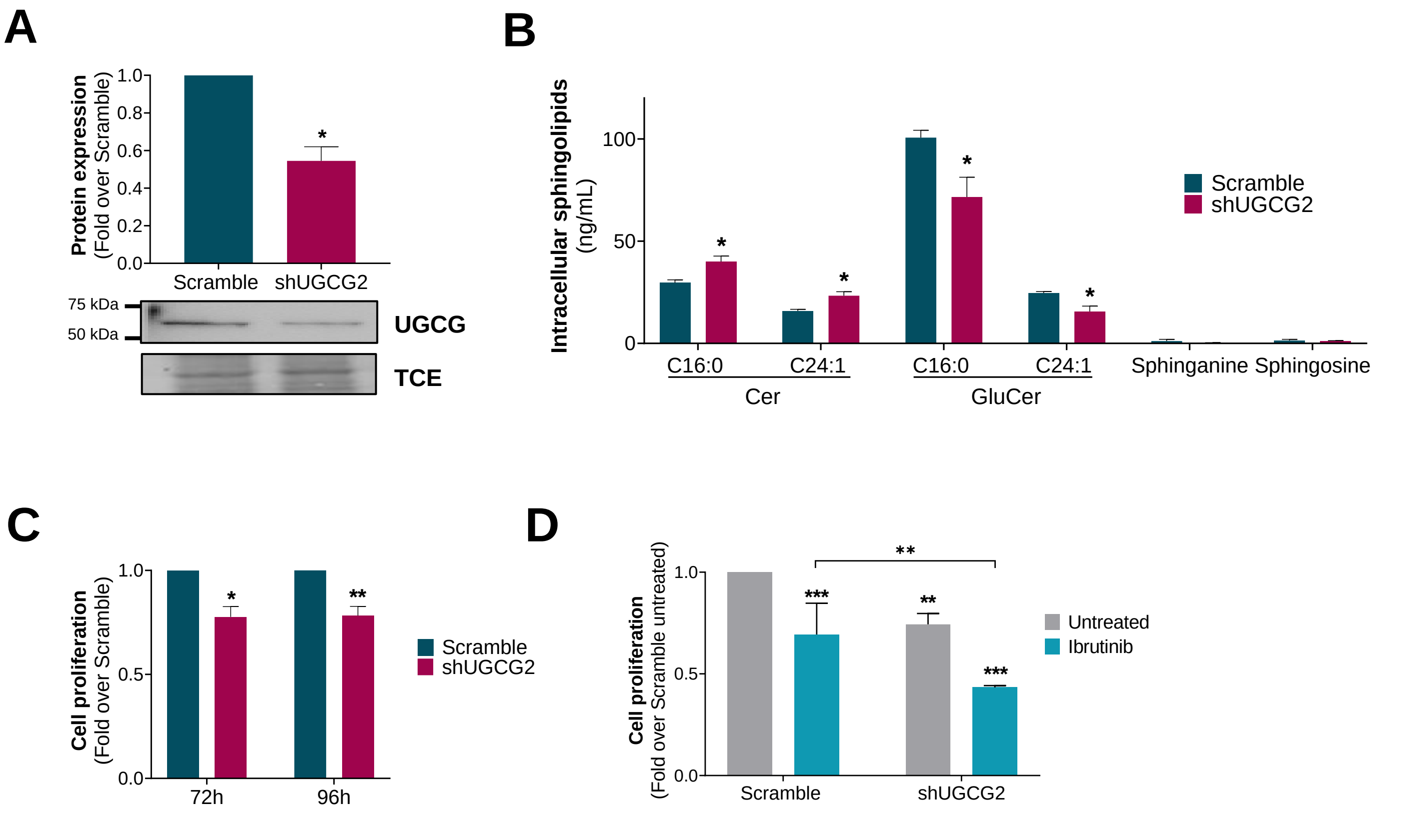

Supplementary Figure 7

Knockdown of UGCG in HG3 was validated at protein levels (A) and UGCG activity (B) by quantifying intracellular sphingolipids. Data are expressed as the mean ± standard error from two independent experiments. (C) Effect of UGCG knockdown on HG3 cell proliferation was assessed by cell counting, cells were seeded at 0.1x10^6 cells/mL in 5 mL and viable cells were counted in every 24h for four days. Cell proliferation was calculated as fold over scramble. (D) HG3 cells were treated with 0.3 µM ibrutinib for 96h and cells were counted. Cell proliferation was calculated as fold over untreated scramble cells. Data are expressed as the mean ± standard error from three independent experiments. *P < .05; **P < .01; ***P < .001. TCE: 2,2,2-trichloro-ethanol

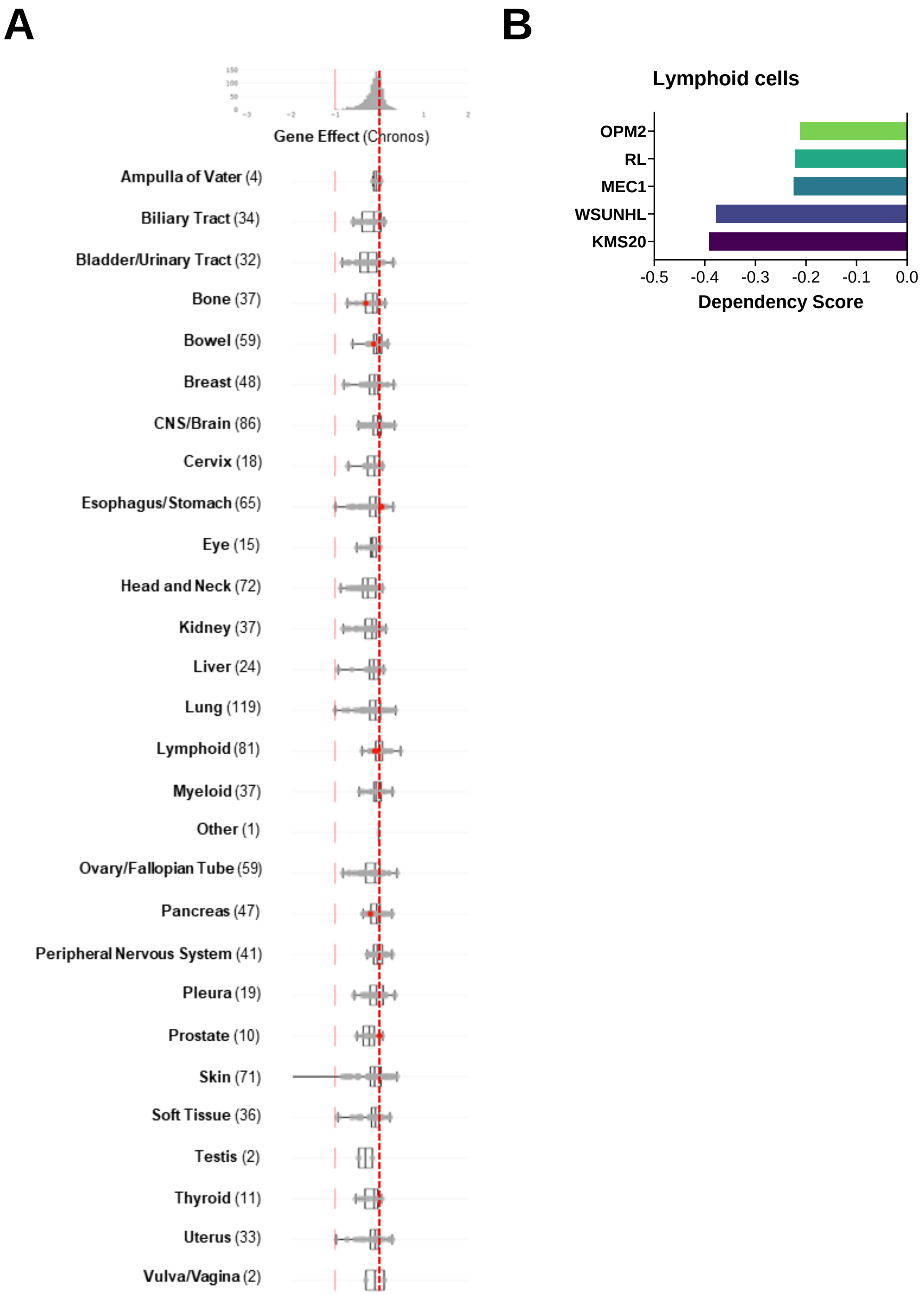

Supplementary Figure 8

(A) Effect of *UGCG* knockout (KO) by CRISPR/Cas9 on cell growth and survival on human cancer cell lines using the DepMap project data (DepMap 22Q2 Public + Score, Chronos). A negative dependency score (Gene Effect, as shown with the red dotted lines) induced by *UGCG* KO corresponds to decrease in cell growth and survival in cancer cell lines. (B) Top 5 most sensitive lymphoid cancer cell lines to *UGCG* KO. Higher the dependency score is negative, higher is the cell sensitivity to *UGCG* KO.

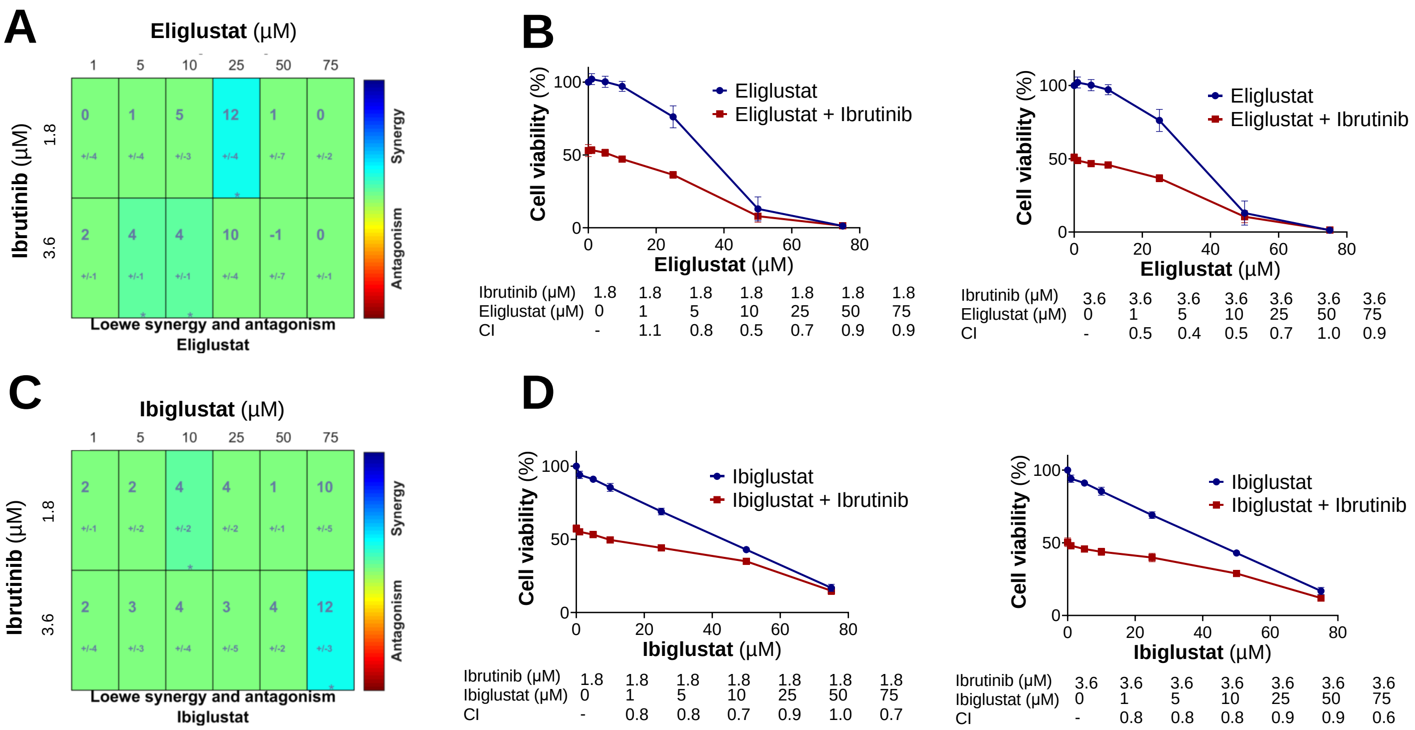

Supplementary Figure 9

HG3 cells were co-treated for 72h with IC_{50} or $IC_{50/2}$ concentrations of ibrutinib (1.8 and 3.6 µM) and increasing concentrations of UGCGi: eliglustat (A-B) and ibiglustat (C-D). Effect of the treatment combination was calculated using the Loewe Additivity model using two softwares: SynergyFinder+ and Combenefit. The synergy score was represented in a heatmap using Combenefit (A,C) and in graphs representing the combination index (CI) using SynergyFinder+ (B,D). With Combenefit, a synergy score > 0 represents a synergy, additive if the score = 0, and antagonistic if the score < 0. With SynergyFinder+, CI < 1 represents a synergy, CI = 1 is additive and >1 is antagonistic

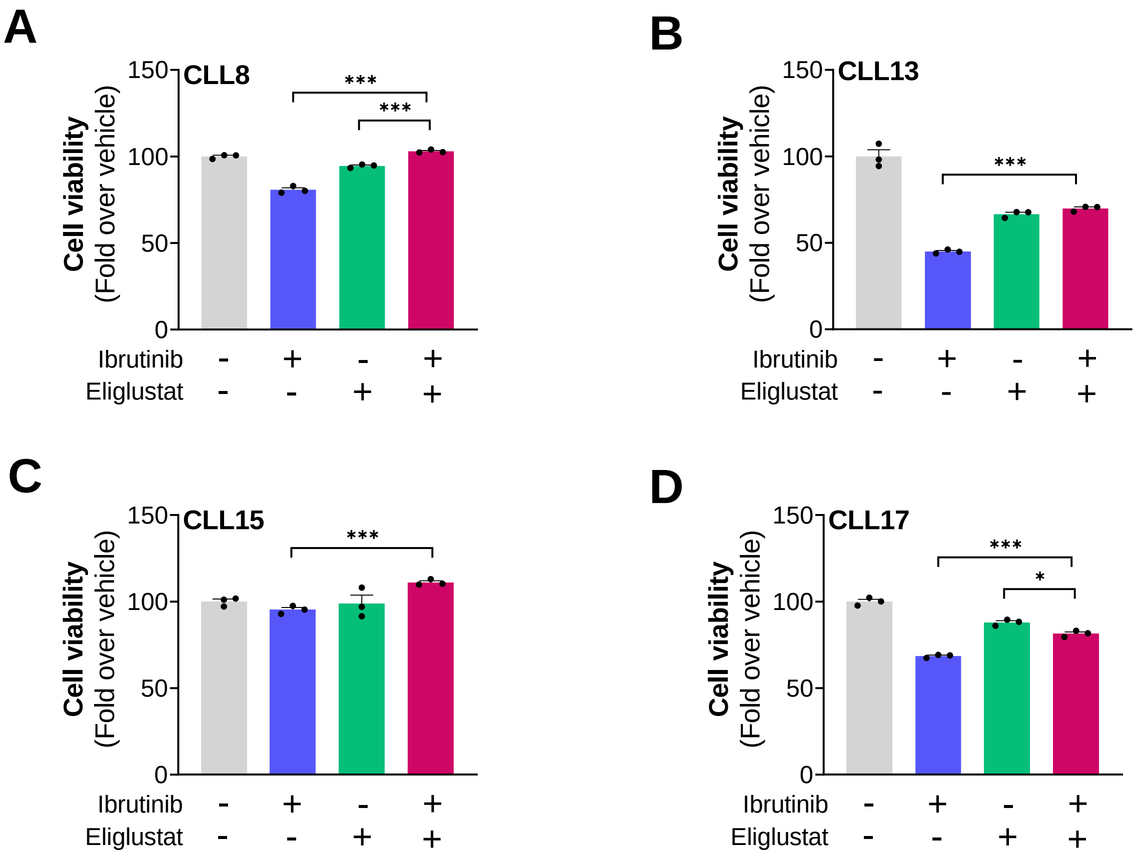

Supplementary Figure 10

(A-D) Primary cells from six CLL patients were treated with vehicle (DMSO), 0.3 µM ibrutinib, 50 µM eliglustat alone and in combination for 46h. Cell viability was measured by CellTiter-blue assay in triplicate for each patient. In this figure is represented CLL patients that were not sensitive to the treatment combination compared to ibrutinib and eliglustat treatments alone. Data are expressed as the mean ± standard error. **P* < .05; ***P* < .01; ****P* < .001.

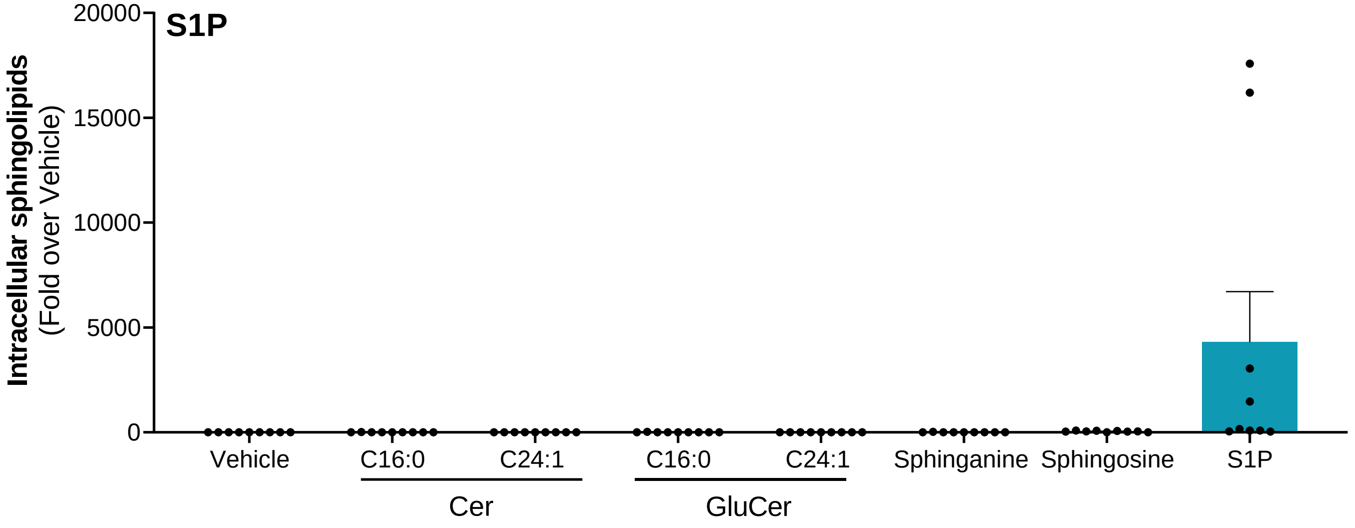

Supplementary Figure 11

Primary cells from CLL patients (n=12) were treated with 10 µM S1P for 22h. 9 CLL patients out of 12 presented an accumulation of intracellular S1P confirmed by mass spectrometry. Data are expressed as the mean ± standard error. *P < .05; **P < .01; ***P < .001. S1P : sphingosine-1-phosphate.

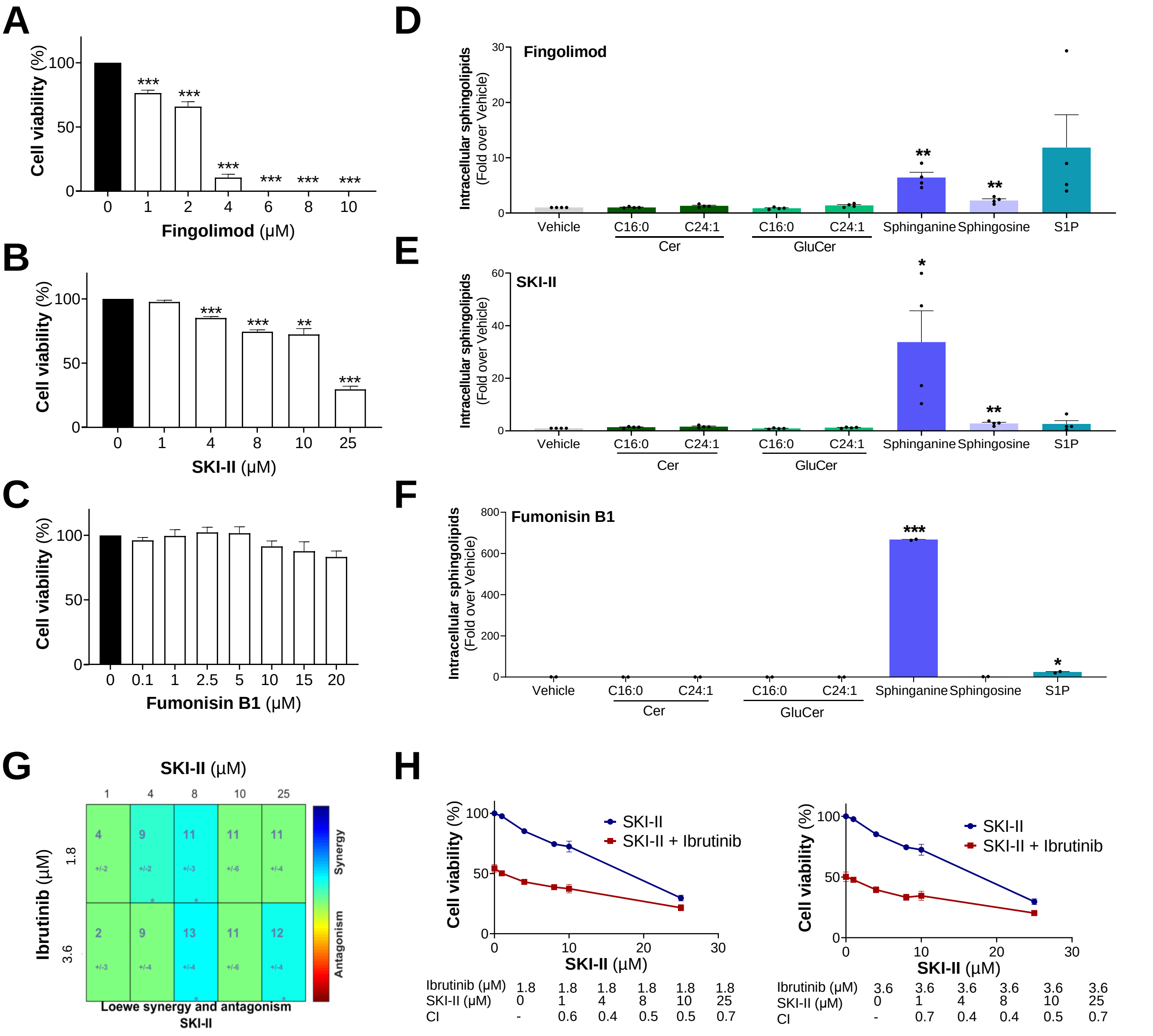

Supplementary Figure 12

HG3 cells were treated with the SPHK inhibitors (SPHKi), fingolimod (A,D) and SKI-II (B,E) and CERS inhibitor (CERSi), fumonisin B1 (C,F) for 72h. (A-C) Effect of SPHKi and CERSi on cell viability was measured by MTS assay at 490 nm and calculated compared to untreated cells. (D-F) Efficacy and specificity of the inhibitors were assessed by quantifying intracellular sphingolipids concentration. HG3 cells were treated with IC_{50} concentrations of SPHKi for 72h corresponding to 1.5 µM fingolimid (D) and 19 µM SKI-II (E) and 20 µM fumonisin B1 (F). (G-H) HG3 cells were co-treated for 72h with IC_{50} or $IC_{50/2}$ concentrations of ibrutinib (1.8 and 3.6 µM) and increasing concentrations of SKI-II. Effect of the treatment combination was calculated using the Loewe Additivity model. The synergy score was represented in a heatmap using Combenefit software (G) and in graphs representing the combination index (CI) using SynergyFinder+ software (H). With Combenefit, a synergy score > 0 represents a synergy, additive if the score = 0, and antagonistic if the score < 0. With SynergyFinder+, CI < 1 represents a synergy, CI = 1 is additive and >1 is antagonistic. Data are expressed as the mean ± standard error from minimum two independent experiments. $^{*}P < .05$; $^{**}P < .01$; $^{***}P < .001$.

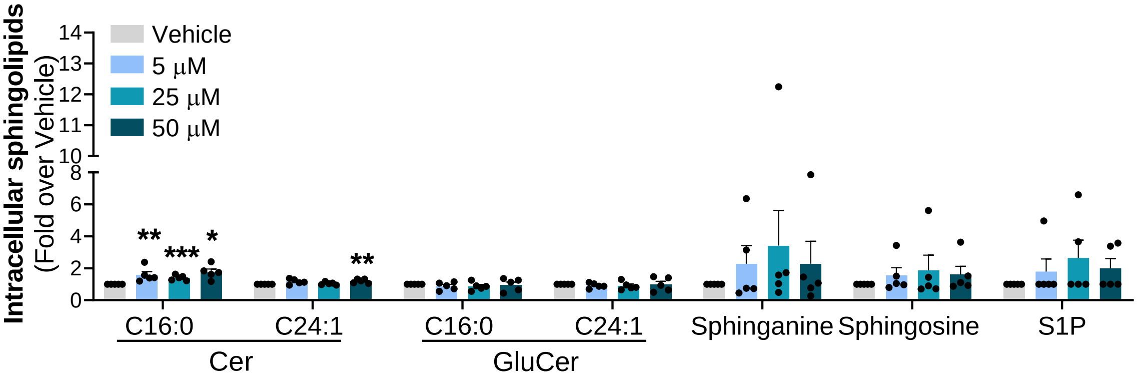

Supplementary Figure 13

Peripheral blood mononuclear cels (PBMCs) from five CLL patients were treated with 5, 25 and 50 µM SKI-II for 46h. Effects of SKI-II treatment were assessed by mass spectrometry. Data are expressed as the mean ± standard error. **P* < .05; ***P* < .01; ****P* < .001. S1P : sphingosine-1-phosphate.

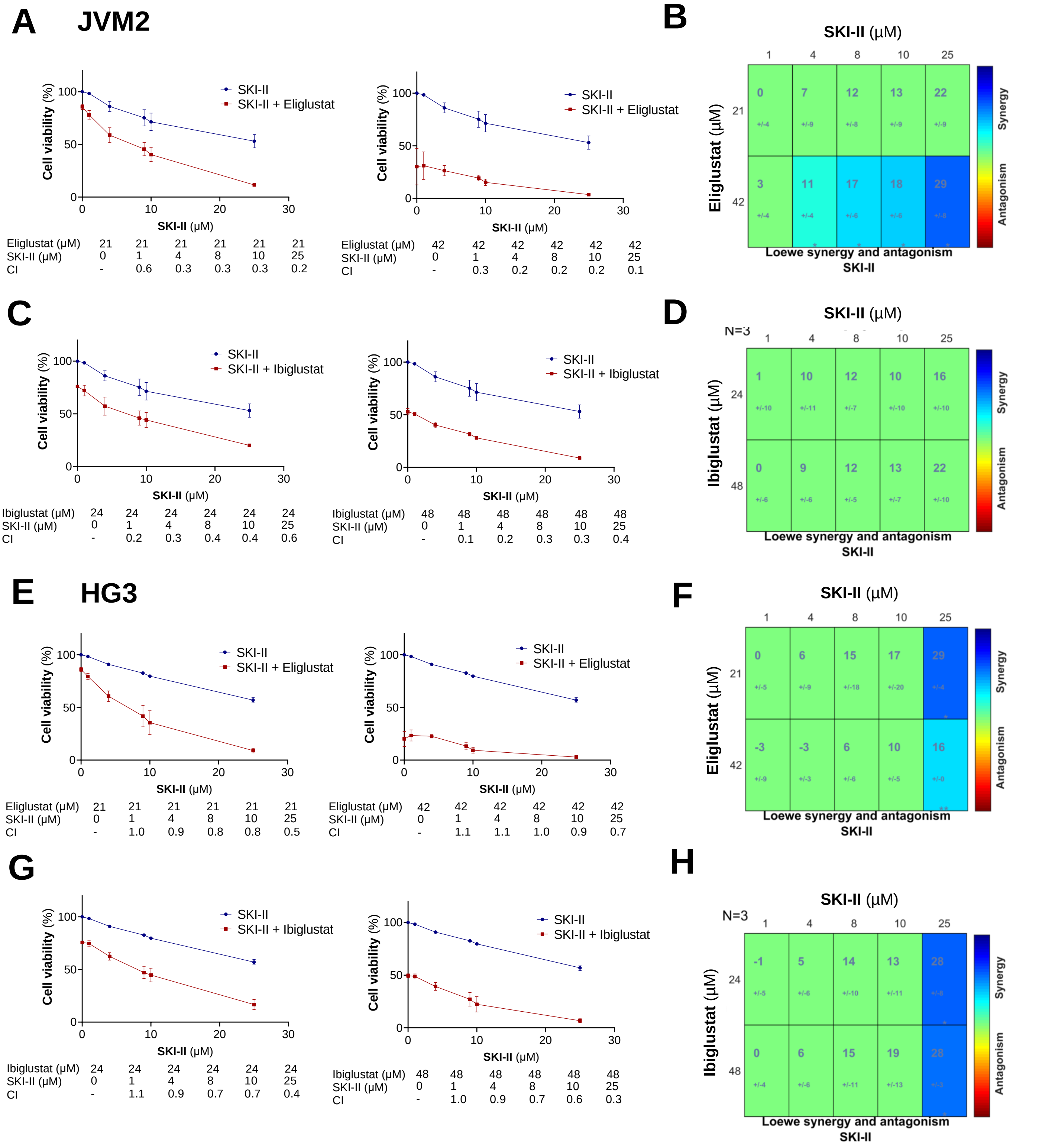

Supplementary Figure 14

JVM2 (A-D) and HG3 cells (E-H) were co-treated for 72h with two concentrations of UGCG inhibitors (UGCGi): 21 and 42 µM eliglustast (A-B; E-F) or 24 and 48 µM ibiglustat (C-D; G-H) and increasing concentrations of the SPHK inhibitor, SKI-II. Effect of the treatment combination was calculated using the Loewe Additivity model using two softwares: SynergyFinder+ and Combenefit. The combination index (CI) was represented in graphs using SynergyFinder+ (A,C,E,G), CI < 1 represents a synergy, CI = 1 is additive and >1 is antagonistic. The synergy score was represented in a heatmap using Combenefit (B,D,F,H), a synergy score > 0 represents a synergy, additive if the score = 0, and antagonistic if the score < 0.
