# Supplementary material for: Targeting sphingolipid metabolism in chronic lymphocytic leukemia
Source: Clin Exp Med. 2024 Jul 30;24(1):174. doi: 10.1007/s10238-024-01440-x (PMC11289351; doi:10.1007/s10238-024-01440-x)
Supplement: Supplementary file 2 — Supplementary file2 (PDF 255 KB) [file 10238_2024_1440_MOESM2_ESM.pdf]

Targeting sphingolipid metabolism in chronic lymphocytic leukemia

Flora Nguyen Van Long[1], Trang Le[2], Patrick Caron[1,] Délya Valcourt-Gendron[1], Roxanne Sergerie[1], Isabelle Laverdière[1], Katrina Vanura[2] and Chantal Guillemette*[1,3]

[1]Centre Hospitalier Universitaire de Québec Research Center - Université Laval (CRCHUQc-UL), Faculty of Pharmacy and Centre de Recherche sur le Cancer (CRC-UL), Université Laval, Québec, Canada

[2]Department of Medicine I, Division of Haematology and Haemostaseology, Medical University of Vienna, Vienna, Austria.

[3]Canada Research Chair in Pharmacogenomics

This file includes:
Supplementary Material and Methods
Supplementary Tables 1-4

Supplementary Material and Methods

Chemicals

Methanol (MeOH), and chloroform were obtained from VWR Canlab (Montreal, QC, Canada). C16 ceramide d18:1/16:0 (C16:0 Cer), C24:1 Ceramide d18:1/24:1 (C24:1 Cer), C24:1 Glucosyl (β) Ceramide d18:1/24:1 (C24:1 GluCer) and C16 glucosyl (β) Ceramide d18:1/16:0 (C16:0 GluCer) were purchased from Avanti Polar Lipids (Alabaster, AL, USA). D-Erythro-sphingosine (d18:1 sphingosine), D-Erythro-C18 Dihydro D-sphingosine (d18:0 sphinganine) and sphingosine-1-phosphate (d18:1-S1P) were purchased from TRC (North York, ON, Canada). Internal standards D-erythro-sphingosine-d7 (d18:1 sphingosine-d7) and C16 ceramide d18:1-d7/16:0 (C16:0 Cer-d7) were purchased from Avanti Polar Lipids. Eliglustat, fingolimod, SKI-II and ibrutinib were purchased from Selleck Chemicals (Houston, TX, USA). Ibiglustat and fumonisin B1 was obtained from Cayman Chemical (Ann Arbor, MI, USA).

Cell models and culture

All cell culture components were purchased from Wisent Bioproducts (St-Bruno, QC, Canada). Cell lines were regularly tested for mycoplasma contamination. The NCBI BioSample database (https://www.ncbi.nlm.nih.gov/biosample) was used to verify that all leukemic cell lines used in this study were not misidentified or contaminated by other human cell lines. JVM2 cells were cultured in RPMI medium supplemented with 10% fetal bovine serum (FBS), 1% penicillin/streptomycin, 1% sodium pyruvate and 1% L-glutamine and 5 µg/mL blasticidin in 5% CO2 at 37°C. HG3 cells were cultivated in same growth media but with 15% FBS and without sodium pyruvate and blasticidin.

For UGCG knockdown in JVM2 and HG3 cells, shRNAs sequences targeting human UGCG are displayed in **Supplementary Table 4**. Lentivirus production and transduction steps were conducted as described previously[1]. Selections of cells containing the shRNAs were performed by adding 0.5 µg/mL puromycin for HG3 cells and 1 µg/mL puromycin for JVM2 cells. Validation of the knockdown was performed by RT-qPCR and immunoblotting for UGCG expression and by measuring intracellular sphingolipid levels for UGCG enzyme activity. shRNA demonstrating sufficient repression of UGCG expression were selected for further functional studies.

Cell-based assays

To assess cell viability, JVM2 and HG3 cells were plated at a density of 5 x 10^4 cells/well and 1 x 10^4 cells/well, respectively, in in 96-well U-bottom tissue culture plates (BD Bioscience, Franklin Lakes, NJ, USA). Cells were treated with various concentrations of sphingolipid inhibitors alone or in combination with ibrutinib. After 72h incubation, 20 µl of CellTiter aqueous one solution cell proliferation reagent (MTS Promega, Madison, WI, USA) was added to each well. Absorbance at 490 nm was measured in the Infinite M1000 plate reader (Tecan, Männedorf, Switzerland) after a 4-h incubation with CellTiter in the cell culture incubator at 37°C. Assays were replicated at least three times, in triplicate.

Apoptosis assays were carried out using 2 x 10^6 cells as previously described[1] using Violet Annexin V/Dead cell Apoptosis Kit (Invitrogen, Eugene, OR, USA). Assays were replicated in biological triplicate.

To assess cell proliferation in cells with *UGCG* knockdown, cells counting was performed for 96h. HG3 and JVM2 cells were plated at a density of 1 x 10^5 cells/mL in T25 flasks (Corning, Durham, NC, USA) in duplicate for each condition. Every 24h, 10 µL of cells were sampled and mixed with

50% of trypan blue (Wisent) and stained cells were counted using a TC-10 automated cell counter in triplicate (Bio-Rad, Hercules, CA, USA). Cell numbers resulted from the average of six values per condition. Cell proliferation assays were replicated at least in a biological duplicate.

Gene and protein expression analyses

To validate UGCG knockdown, UGCG expression was quantified by RT-qPCR and immunoblotting. RNA extraction, reverse transcription and qPCR were performed as previously described[1]. Relative expression levels were calculated using the 2–ΔΔCT method and normalized with 36B4 as the reference gene. Primer sequences are provided in Supplementary Table 3.

For protein expression, 20 x 10^6 cells were collected, washed and then lysed in lysis buffer consisting of 150 mM NaCl, 50 mM Tris-HCl (pH 7.4), 0.3% sodium deoxycholate, 1% IGEPAL CA-630 (Sigma- Aldrich, Burlington, MA, USA), 1 mM EDTA, protease and phosphatase inhibitors (Sigma-Aldrich). Protein quantification and immunoblotting were performed as described previously[1]. Primary antibody directed against UGCG (#12869-1-AP) was diluted at 1:600 and purchased from Proteintech (Rosemont, IL, USA). The secondary antibody was a horseradish peroxidase–linked anti–rabbit IgG from donkey and was diluted at 1:10 000 (#45000682; Thermo Fisher Scientific, Rockford, IL, USA). As the loading control, total protein stain (2,2,2-trichloro-ethanol; Sigma-Aldrich, 1:200) was used. For western blot images, one representative loading control was presented. Normalization of each protein expression was performed using each loading control that was on the same membrane as the protein quantified.

Cancer cell lines dependency on *UGCG*

UGCG dependency data by CRISPR/Cas9 system were issued from the Public 23Q4 dataset released by the DepMap project from Broad Institute. The data was processed using Chronos algorithm3. Data are available online at https://depmap.org/portal/ and UGCG dependency analyses across cancer cell lines were performed using the "Perturbation Effects" online tool.

Supplementary Table 1. Characteristics of 17 CLL patients assessed in this study

ID	Age	Sex	IGHV	CD38	Binet	Del11q	Tris 12	Del13q	P53	Treatments
CLL1	62.8	F	NA	High	A	Yes	No	No	UM	Extracellular sphingolipids
CLL2	58.3	M	UM	High	A	No	No	No	UM	Extracellular sphingolipids
CLL3	47.2	M	M	High	A	Yes	No	Yes	UM	Extracellular sphingolipids
CLL4	57.7	M	M	Low	A	No	No	Yes	UM	Extracellular sphingolipids
CLL5	45.6	M	UM	High	B/C	Yes	No	No	UM	Extracellular sphingolipids
CLL6	63.2	M	M	Low	B/C	No	No	Yes	UM	Extracellular sphingolipids
CLL7	62.5	M	M	High	A	No	Yes	No	UM	Extracellular sphingolipids
CLL8	54.1	F	M	Low	A	No	No	No	UM	Extracellular sphingolipids<br>Combination with ibrutinib
CLL9	43.9	M	UM	Low	B/C	No	No	Yes	UM	Extracellular sphingolipids
CLL10	64.0	M	M	Low	A	No	No	Yes	UM	Extracellular sphingolipids
CLL11	64.0	M	M	Low	A	No	No	Yes	UM	Extracellular sphingolipids
CLL12	47.5	F	M	Low	A	No	No	Yes	UM	Extracellular sphingolipids
CLL13	52.9	M	UM	Low	A	Yes	No	Yes	UM	Sphingolipid inhibitors<br>Combination with ibrutinib
CLL14	39.9	F	M	High	A	No	Yes	No	UM	Sphingolipid inhibitors<br>Combination with ibrutinib[$]
CLL15	64.1	F	NA	Low	A	No	No	Yes	UM	Sphingolipid inhibitors<br>Combination with ibrutinib[$]
CLL16	25.3	F	UM	High	A	Yes	No	No	UM	Sphingolipid inhibitors<br>Combination with ibrutinib
CLL17	69.6	M	NA	High	A	Yes	No	Yes	M	Sphingolipid inhibitors<br>Combination with ibrutinib

F: female; M: male; M: mutated; M: unmutated; Tris 12: trisomy 12; Del11q: 11q chromosome deletion; Del13q: 13q chromosome deletion. [$]Patients that were sensitive to the combination of ibrutinib and UGCG inhibitor, eliglustat based on the significant reduction of cell viability by the drug combination compared to both drugs treatments alone.

Supplementary Table 2. Characteristics of CLL patients assessed in the evaluation of sphingolipid inhibitors alone and in combination with ibrutinib on cell viability.

	**PBMC (N=6)**	
	**N**	**(%)**
**Age**		
(median, min-max years)	53.5 (25.3 – 69.6)	
**Sex**		
Female	4	(67)
Male	2	(33)
**IGHV mutational status**		
N/A	2	(33)
Mutated	2	(33)
Unmutated	2	(33)
**Binet Stage**		
N/A		
A	6	(100)
B or C		
**CD38 expression**		
N/A		
Low	3	(50)
High	3	(50)
**Del11q**		
N/A		
Negative	3	(50)
Positive	3	(50)
**Trisomy 12**		
N/A		
Negative	5	(83)
Positive	1	(17)
**Del13q**		
N/A		
Negative	3	(50)
Positive	3	(50)
**P53 mutation**		
N/A		
Negative	5	(83)
Positive	1	(17)

PBMC: peripheral blood mononuclear cells; N/A: data not available; Del11q: 11q chromosome deletion; Del13q: 13q chromosome deletion

Supplementary Table 3. Characteristics of CLL patients assessed in the evaluation of extracellular sphingolipid treatments on CLL cell survival.

	**PBMC (N=12)**	
	**N**	**(%)**
**Age**		
(median, min-max years)	58.0 (47.2 – 64.0)	
**Sex**		
Female	3	(25)
Male	9	(75)
**IGHV mutational status**		
N/A	1	(8.3)
Mutated	8	(66.7)
Unmutated	3	(25)
**Binet Stage**		
N/A		
A	9	(75)
B or C	3	(25)
**CD38 expression**		
N/A		
Low	7	(58.3)
High	5	(41.7)
**Del11q**		
N/A		
Negative	9	(75)
Positive	3	(25)
**Trisomy 12**		
N/A		
Negative	11	(91.7)
Positive	1	(8.3)
**Del13q**		
N/A		
Negative	5	(41.7)
Positive	7	(58.3)
**P53 mutation**		
N/A		
Negative	12	(100)
Positive		

PBMC: peripheral blood mononuclear cells; N/A: data not available; Del11q: 11q chromosome deletion; Del13q: 13q chromosome deletion

Supplementary Table 4. Sequences of shRNAs targeting *UGCG* and primers used in this study.

**shRNA names**	**Target sequence (5'-3')**	
shUGCG1	GCAGAGGAAATCCTAGATGTA	
shUGCG2	CCGCGAATCCATGACAATATA	
shUGCG3	GTGGACCAAACTACGAATTAA	
shUGCG4	GCATTATGGGACCCAACTATA	
**qPCR**	**Forward sequence (5' – 3')**	**Reverse sequence (5' – 3')**
36B4 (RPLP0)	CCCATGTGAAGTCACTGTGC	GGTTGTAGATGCTGCCATTG
UGCG	TGCTCAGTACATTGCCGAAG	TTCGTAGTTTGGTCCACCTG

References for Supplementary Material and Methods

1. Nguyen Van Long F, Valcourt-Gendron D, Caron P, et al. Untargeted metabolomics identifies metabolic dysregulation of sphingolipids associated with aggressive chronic lymphocytic leukaemia and poor survival. *Clin Transl Med*. 2023;13(12):e1442. doi:10.1002/ctm2.1442
